# Adiabatic Connection Correlation Functionals in Metallic Solids from Hartree–Fock Gaussian Basis Set Ground State

**DOI:** 10.1021/acs.jpclett.6c00976

**Published:** 2026-06-10

**Authors:** Fabio Della Sala, Fulvio Sarcinella, Lucian A. Constantin, Lorenzo Donà, Eduardo Fabiano, Lorenzo Maschio, Bartolomeo Civalleri

**Affiliations:** † Institute for Microelectronics and Microsystems (CNR-IMM), Via Monteroni, Campus Unisalento, 73100 Lecce, Italy; ‡ Department of Chemistry, 9314 University of Turin, via Giuria 7, 10125 Torino, Italy; § Center for Biomolecular Nanotechnologies, Istituto Italiano di Tecnologia, Via Barsanti 14, 73010 Arnesano, Lecce, Italy

## Abstract

Adiabatic Connection Integrand Interpolation (ACII) methods, which mix the Görling-Levy second-order perturbation theory (GL2) correlation with density functionals from the strong interaction regime, have been successfully applied to molecular systems, strongly correlated systems, as well as the uniform electron gas (UEG). Here, we consider real metallic solids, including transition metals, where the GL2 term diverges. We tested different ACII approaches, with increased accuracy in reproducing the UEG correlation, and with different strong interaction functionals within the Point-charge-plus-Continuum (PC) model. Calculations are based on the Hartree–Fock (HF) ground state, which has been obtained with a Gaussian Type Orbital basis set, properly reoptimized toward the HF global minimum, with a derivative-free approach introduced here. We compare different metallic properties such as lattice constants, cohesive energies, and bulk total correlation energies with accurate reference results and state-of-the-art Density Functional Theory (DFT) approaches. We found that only the ACII approach with high accuracy for the UEG, namely, the Density Parameter Interpolation (DPI), and with the strong interaction functional reproducing the exact PC model for the Wigner crystal yields a correct second-order gradient-expansion correlation coefficient. This allows reproducing lattice constants and bulk correlation energies with an accuracy comparable to the best state-of-the-art approaches. When DPI is combined with GL2 correlation of atoms, accurate cohesive energies are obtained without error cancellation, which is common in DFT methods.

## Graphical abstract

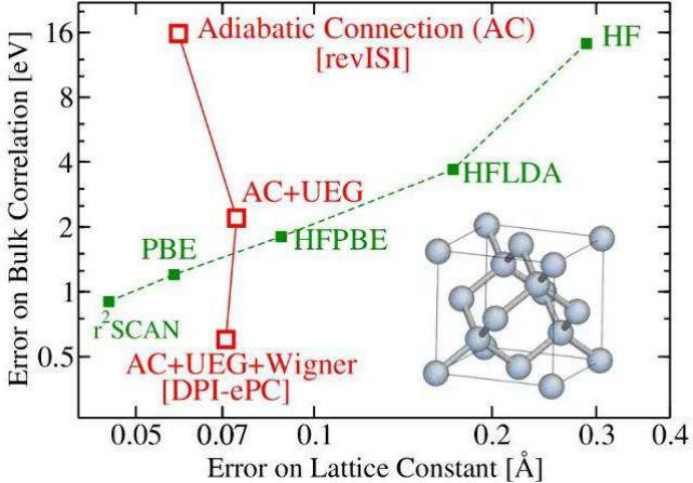

Electronic structure calculations of finite and extended systems can be efficiently and accurately computed within the Kohn–Sham (KS) Density Functional Theory (DFT).^1–6^ The main ingredient of KS-DFT is the ground state electronic density *n*(**r**), which implicitly determines all the ground state properties of the electronic system.^7,8^ KS-DFT is exact, in principle, but the exchange-correlation (XC) energy functional *E*_*xc*_[*n*(**r**)] is unknown and must be approximated.

An elegant (and exact, in principle) definition of the XC functional uses the adiabatic connection (AC) method:^9–15^

1 $$\begin{array}{lll} E_{xc}\left[n\right] & = & \int_{0}^{1}d\alpha W_{xc,\alpha }\left[n\right]\\ W_{xc,\alpha }\left[n\right] & = & \left\langle \Psi_{n}^{min,\alpha }\right|{\mathrm{V̂}}_{ee}|\Psi_{n}^{min,\alpha }]\rangle -U\left[n\right] \end{array}$$

where ${\mathrm{V̂}}_{ee}$ is the Coulomb repulsion operator, and $\Psi_{n}^{min,\alpha }$ is the antisymmetric wave function that yields the density *n*(**r**) and minimizes the expectation value $\left\langle \mathrm{T̂}+\alpha {\mathrm{V̂}}_{ee}\right\rangle$ , with α ≥ 0 being the coupling constant (also known as interaction strength). Different XC functionals have been constructed in the framework of the AC method, including accurate hybrid functionals^7,14,16^ and the most sophisticated fifth-rung functionals^17^ (which use the unoccupied orbitals in the functional definition) such as the random phase approximation (RPA)^18–20^ and double-hybrid (DH) functionals.^21^

In this work, we consider the Adiabatic Connection Integrand Interpolation (ACII) methods,^22–54^ which also belong to the fifth rung. Pioneering ACII approaches are the Interaction Strength Interpolation (ISI)^23^ and the revised ISI (revISI).^27^ Recently, more accurate ACII functionals, and with different extensions, have been presented.^43,44,46^ ^,^ ^47,49–53^

The ACII methods are based on a *W*_*xc*,α_[*n*] model which interpolates between the weak (α → 0) and strong (α → *∞*) interaction limits, which are known exactly:^23,28,55^

2 $$W_{xc,\alpha \to 0}\left[n\right]=E_{x}^{\mathrm{EXX}}\left[n\right]+2E_{c}^{GL2}\left[n\right]\alpha +...$$

3 $$W_{xc,\alpha \to \infty }\left[n\right]=W_{\infty }\left[n\right]+W_{\infty }^{\prime }\left[n\right]\alpha^{-1/2}+...$$

Here, *E*_*x*_[*n*] is the exact DFT exchange functional and $E_{c}^{GL2}\left[n\right]$ is the second-order term of the Görling-Levy perturbation theory (GL2).^55,56^ In some schemes, the GL2 terms are approximated with a semilocal functional:^12,42,47,48^ in this way, however, the whole functional no longer belongs to the fifth rung.

The quantities *W*_*∞*_[*n*] and $W_{\infty }^{\prime }\left[n\right]$ can be computed using the strictly correlated electron (SCE) approach^57–60^ or, more efficiently, approximated using generalized gradient approximation (GGA) or meta-GGA formulas.^24,40,43^ ^,^ ^48,54^

Thus, the general expression for the XC energy of ACII functionals is

4 $$E_{xc}^{\mathrm{ACII}}=E_{x}^{\mathrm{EXX}}+F\left(E_{x}^{\mathrm{EXX}},E_{c}^{GL2},W_{\infty },W_{\infty }^{\prime }\right)$$

where F is a nonlinear function, and, for simplicity, we do not show, here and in the following, the density dependence for all quantities.

An interesting limit of the ACII approaches is the one in which $E_{c}^{GL2}\to -\infty$ , such that the XC energy can be written as^43,47^

5 $$\underset{E_{c}^{GL2}\to -\infty }{\lim}E_{xc}^{\mathrm{ACII}}=E_{x}^{\mathrm{EXX}}+G\left(E_{x}^{\mathrm{EXX}},W_{\infty },W_{\infty }^{\prime }\right)$$

where G is another general nonlinear and analytical function. This limit is present in all systems with a vanishing energy gap, such as dissociation of molecules in a restricted formalism or in metallic solids.^43,47,49^ The former has been investigated in different works,^42,43,49^ whereas the latter have been considered only for the uniform interacting electron gas (UEG), also often called jellium, one of the most important model systems for solid state physics.^1,2,8,61^ Recently, some of the authors proposed the genISI^47^ and genISI2 ^49^ XC functionals, both of which have been constructed taking into account the UEG as the main reference system. Thus, in the limit $E_{c}^{GL2}\to -\infty$ , these functionals recover the uegISI functional^47^ which is accurate for the UEG correlation energy per particle. On the other hand, popular ACII XC functionals, such as ISI and revISI, strongly overestimate (in absolute value) the UEG correlation energy per particle.^47^

In this work, we assess the ACII methods for real bulk metals, considering simple (Al), alkali (Li, Na, K), as well as transition metals (Cu, Ag, Au). This is an important test, because, so far, the limiting formula in eq 5 has been tested only on UEG and on the H_2_ dissociation in spin-restricted formalism.^43,47,49^

Metallic solids can be well described by the semilocal functionals (GGA and meta-GGA),^62,63^ being even better than the popular Perdew–Burke–Ernzerhof (PBE0) and Heyd-Scuseria-Ernzerhof (HSE06) hybrid functionals,^64,65^ that may have larger errors for bulk moduli and cohesive energies.^66^ The description of the equilibrium properties of bulk metals represents a hard test for the fifth-rung functionals, because of the vanishing band gap feature. Thus, the second order of the Møller–Plesset perturbation theory (MP2) is also diverging, such that all DH functionals^21^ cannot be applied to these systems. Other sophisticated and expensive methods, such as the Fermi-hypernetted chain approximation, overestimates the UEG correlation energies and the jellium surface XC energies,^67,68^ while the inhomogeneous Singwi-Tosi-Land-Sjölander (ISTLS) is accurate for jellium.^68,69^ On the other hand, the RPA is remarkably accurate for metals,^62^ while the UEG based XC kernels, used for the ground state calculations of linear-response time-dependent DFT, can improve the RPA underestimation of correlation energy, preserving its good performance for bulk metals.^70^ Another approach to avoid the MP2 divergence is to use renormalized denominators.^71^

More recently, state-of-the-art coupled-cluster single–double (CCSD) approaches and their extensions have been successfully applied to metallic solids,^72–77^ defining new reference data.

In this work we describe bulk systems using a Gaussian Type Orbital (GTO) basis set approach. The main motivation is to have a unique framework which allows also to efficiently consider MP2 and CCSD­(T)^78^ approaches. On the other hand, properly optimized GTO basis sets for bulk are required. Existing basis sets^79,80^ are not optimized for all bulk metallic systems under investigation in this work (Li, Na, K, Al, Cu, Ag, Au), and previous schemes,^79^ despite being efficient, do not allow to reach the global minimum. Here, we also present the Discrete Global Basis set optimization (DGBO) approach, which is a derivative-free method for the global minimization, and we will show that accurate GTO basis sets for all metals can be obtained.

## General Theoretical and Computational Framework

To treat metallic systems, we consider the ACII approach in the limit $E_{c}^{GL2}=-\infty$ . In this case the correlation integrand can be easily written as

6 $$W_{c,\alpha }=-\left(E_{x}^{\mathrm{EXX}}-W_{\infty }\right)f_{c}\left(x\right)$$

7 $$x=q\sqrt{\alpha }$$

8 $$q=\frac{E_{x}^{\mathrm{EXX}}-W_{\infty }}{W_{\infty }^{\prime }}>0$$

where *f*_*c*_ ∈ [0, 1] depends on the ACII formula. The total correlation energy is

9 $$E_{c}^{\mathrm{ACII}}=\int_{0}^{1}W_{c,\alpha }d\alpha =-\left(E_{x}^{\mathrm{EXX}}-W_{\infty }\right)F_{c}\left(q\right)$$

10 $$=G\left(E_{x}^{\mathrm{EXX}},W_{\infty },W_{\infty }^{\prime }\right)$$

11 $$F_{c}\left(q\right)=\int_{0}^{1}f_{c}\left(q\sqrt{\alpha }\right)d\alpha$$

and we also have that *F*_*c*_ ∈ [0, 1].

In this work, we considered the four main approximations available in literature, namely: ISI,^23^ revISI,^27^ uegISI^47^ and the Density Parameter Interpolation (DPI).^47,81^ Note that uegISI reproduces the UEG limit accurately (but for small *r*_*s*_ values), whereas ISI and revISI are quite bad.^47^ The DPI functional reproduces exactly the UEG limit for all *r*_*s*_.^47,81^ The full expressions can be found in ref 47 and in section S1.

The first important issue for ACII calculations is related to ground state orbitals. ACII is a KS formalism, thus calculations can be done self-consistently,^43^ but this procedure is computationally quite cumbersome. A typical and accurate approximation is to use EXX orbitals.^49^ Using full EXX for all functionals will give common reference orbitals, among different ACII functionals, so that these can be compared directly, without discussing density-driven errors.^82,83^ For bulk systems, as we consider the metallic limit, we do not need orbitals but only the ground state electronic density. Thus, instead of EXX, the calculations have been done with HF, which gives de facto the same density as EXX.^84^ So, within these considerations, the total ACII energy is

12 $$E_{\mathrm{ACII}}^{bulk}=E_{HF}+G{\left(E_{x}^{HF},W_{\infty },W_{\infty }^{\prime }\right)}_{@HF}$$

where *E*_HF_ is the total HF SCF energy and all ingredients are computed at the HF density. eq 12 indicates that ACII methods for metallic bulk are functionals of the exact-exchange and of (meta-) GGA strong interaction ingredients, and thus are similar (from a computational cost point-of-view) to hybrid-DFT functionals. Yet, $E_{x}^{HF}$ enters in eq 12 in a *nonlinear* way, in contrast to conventional global hybrid functionals.

Periodic calculations at the Hartree–Fock level might be quite cumbersome for GTO basis sets,^85–88^ but in this work we use the CRYSTAL23 code^89,90^ in which the HF is efficiently implemented^91^ (see Section S2 for all the parameters of the calculations).

Considering that Ag and Au can only be done with pseudopotentials (see Table S1), we describe all systems without the core electrons, so that all systems are treated in the same way. This also allows us to compare with previous results in literature for the bulk correlation energies.^72,75,92,93^ Moreover, we found that the ACII results in the bulk are quite sensitive to the treatment of the core region, and thus we postpone discussions on this topic to future investigations.

## Basis Set

For the optimization of the GTO basis sets, we developed a mixed bash/python script which interfaces between the CRYSTAL23 code and the ZOO method.^94,95^ The Discrete Global Basis set Optimization (DGBO)^96^ script reads the CRYSTAL23 input file and performs a global optimization of the exponents. The optimization is performed within a finite number of exponents, using a uniform grid with spacing 10^–*d*^, where *d* = 1 is used for the first guess, *d* = 2 for the main run to select exponents, and *d* = 3 is only for a final refinement.

DGBO minimizes^79,80^

13 $$E_{cond}=E_{HF}+\gamma \log\left(\epsilon_{max}/\epsilon_{min}\right)$$

where ϵ_min_ (ϵ_max_) is the minimum (maximum) eigenvalue of the overlap matrix at the Γ point at the integral reference lattice (IRL) constant (see Table S1). Since there could be different sets of exponents leading to a similar total energy, the γ helps to select exponents with a less critical overlap matrix.^79,80^ The value of γ is set to 0.001 for all systems. This is, de facto, the only input parameter for the basis set optimization. A DGBO run with 7–9 exponents to be optimized lasts about 3–4 weeks, as several thousands of HF runs are required. The basis set is of triple-ζ quality (with some modifications for the solid state), and it is optimized for each system, separately, toward the HF global minimum. The basis set is optimized at the experimental lattice constant. The final basis set is named DGBO-MTZ (which stands for modified triple-ζ). The details of the DGBO-MTZ basis set are reported in Table S1  and discussed in detail in Section S3. The convergence of the DGBO-MTZ is discussed in Section S7. The complete DGBO-MTZ basis sets in the CRYSTAL23 format are reported in Section S10; the corresponding band-structures and density-of-states are reported in Section S11.

## Strong Interaction Functionals

The strong interaction quantities can be computed within a semilocal approximation, namely the Point-charge-plus-Continuum (PC) model^23,24,27^

14 $$W_{\infty }^{PC}\approx \int d^{3}rA\rho^{4/3}+B\frac{|\nabla \rho {|}^{2}}{\rho^{4/3}}$$

15 $$W_{\infty }^{\prime PC}\approx \int d^{3}rC\rho^{3/2}+D\frac{|\nabla \rho {|}^{2}}{\rho^{7/6}}$$

where *A* = – 1.4508, *B* = 0.005317, *C* = 1.535, *D* = –0.028957. We note that there have been different generalized gradient approximation extensions to the PC model,^40,43^ mainly to model rapidly varying regions and/or to perform SCF calculations. In this work, we considered the accurate hPC functional.^43^

The parameter *D* in eq 15 has been initially fixed to *D* = –0.02558 from a meta-generalized gradient approximation value^24^ of the He atom, and after that has been revised to *D* = –0.028957, in ref 27, and it is the one considered in this work. However, we recall that the true gradient expansion of the PC model gives *D* = 0.0197, a quite different and *positive* value.^24^ Thus, *D* is quite a critical parameter which has been fixed from small atoms, such that the extraction of a gradient expansion coefficient may be influenced by the rapidly varying density regions (where a gradient expansion may fail). In this work, we consider very different systems from the isolated atoms, so that the exact *D* coefficient can be important. A strong interaction functional (named ePC) with *D* = 0.0197 has been very recently developed by some of us.^54^ This is a meta-GGA functional and it will be also considered in the present work. In particular, the ePC model has been shown to work well with model densities resembling the ones with pseudopotentials, thus good results can be expected for the metallic systems investigated in this work.

## Reference Atoms

To compute the cohesive energy of a bulk system, the total energy of the isolated atom is also required. The cohesive energy curve of bulk system is defined as

16 $$e\left(L\right)=E^{bulk}\left(L\right)-E^{atom}$$

where *E* ^atom^ is the total energy of isolated atom in the free-space and *E* ^bulk^(*L*) is the total energy of the bulk with *L* as the lattice constant. The definition in eq 16 is valid for a complete basis set (e.g., in a plane-wave calculation). When the basis set is finite (e.g., for conventional GTO basis sets), one needs use the Boys–Bernardi counterpoise correction^85,97^ in order to remove the basis set superposition error (BSSE) in the prediction of the bulk properties. The BSSE corrected cohesive energy is given by

17 $$e^{BSSE}\left(L\right)=E^{bulk}\left(L\right)-E^{ghost}\left(L\right)$$

where the calculation of *E* ^ghost^(*L*) refers to the atom surrounded by shells (3 in this work) of ghost atoms located at the same position of the atoms in the lattice. For a small basis set, the two curves in eq 16 and eq 17 can be quite different, whereas they should coincide for a complete basis set (see Section S6  for further details).

The calculation of cohesive energies, which is straightforward in conventional DFT, is more cumbersome for ACII approaches. In ACII, the cohesive energy is computed as the difference between two different approaches: a DFT-like functional (as *W*_*∞*_ and $W_{\infty }^{\prime }$ are simple semilocal DFT functionals) for the bulk and double-hybrid-like functional for the atom, see eq 4, as in isolated atom the $E_{c}^{GL2}$ is finite). Thus, no error cancellation can be present and all quantities must be computed very accurately.

The calculation of atomic correlation energies with ACII functionals is not simple, due to the calculation of the GL2 term. In fact, the GL2 correlation must be computed accurately, using large basis sets as well as a complete basis set (CBS) extrapolation, since the GL2 correlation is strongly basis set dependent^34,49^ Even if the DGBO-MTZ basis set augmented with the surrounding ghost functions is well converged for ground state properties, the calculation of GL2 correlation energies is particularly sensitive to the description of the virtual orbital space. Thus, very large basis sets including high angular-momentum functions are required to achieve convergence.^99–102^ Moreover, for finite systems the GL2 energy strongly depends on orbitals.^49^ A quite accurate approach is to use EXX orbitals, which are quite close to the exact or self-consistent ones. Thus, the EXX orbitals are used for both bulk and atomic systems.

The total energy for the atom can be computed as (see section S6):

18 $$E_{\mathrm{ACII}}^{atom}\approx E_{HF}+F{\left(E_{x}^{HF},E_{c}^{GL2/CBS@\mathrm{EXX}},W_{\infty },W_{\infty }^{\prime }\right)}_{@HF}$$

where all quantities are evaluated at HF orbitals but the GL2 term which uses EXX orbitals. eq 18 indicates that ACII methods for atoms are functionals of the exact exchange, the GL2 correlation and (meta-) GGA strong interaction ingredients, and thus are similar (from a computational cost point-of-view) to DH functionals. Yet, both $E_{x}^{HF}$ and $E_{c}^{GL2/CBS@\mathrm{EXX}}$ , enters in eq 18 in a *nonlinear* way, in contrast to conventional DH functionals.

The correlation energies of isolated atoms for all methods as well as the CCSD­(T)/CBS reference have been computed using the TURBOMOLE program package.^103,104^ The procedure for the CBS extrapolation is described in Section S4. The CCSD­(T)/CBS results are reported in the last column of Table 1. For K, Al, and Cu our results are fully in agreement with previous calculations.^98^

**Table 1:**
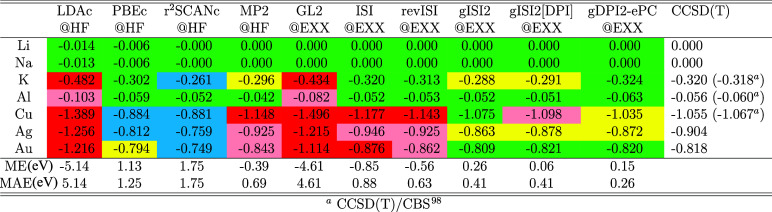
Correlation Energies (Valence-Only) at the CBS Limit for All Atoms and Methods, in a.u.[Table-fn tbl1-fn1]

b ACII methods and GL2 use EXX orbitals, while the other ones use HF orbitals. gISI2 is a shorthand for genISI2, gDPI2 is a shorthand for genDPI2. The last two lines report the mean error (ME) and the mean absolute error (MAE) in eV. Cell colors represent errors E in a.u. with respect to CCSD­(T): red (*E* < – 0.05), pink (−0.05 < *E* < – 0.02), green (−0.02 < *E* < + 0.02), yellow (+0.02 < *E* < + 0.05), cyan (*E* > + 0.05).

We consider the ISI, revISI, and genISI2 ^49^ expression which generalizes uegISI including the GL2 correlation, see the general expression in eq 4. For DPI we use the same genISI2 functional, but with the uegISI replaced with the DPI (named genISI2­[DPI]), or the genDPI2 functional introduced here, which is constructed similarly to the genISI2 functional, yet with some key differences: details are reported in Section S4.a  and will be further discussed in a forthcoming publication. Note that we always used the hPC strong interaction functional, but for genDPI2 which uses ePC. A detailed comparison of these functionals have been presented in ref 49. for different systems, and genDPI2-ePC works as genISI2 (or better). Recall that the genISI2/genDPI2 functionals only enter in the calculation of atomic correlation energies and bulk cohesive energies, whereas for all the other bulk properties discussed in this work (i.e., lattice constants, bulk moduli and bulk correlation energies) are only based on uegISI/DPI.

In Table 1, we report the valence-only correlation energies (with sign) for all atoms and methods. All values are extrapolated to the CBS limit. LDAc, PBEc, and r^2^SCANc, which indicate the correlation part of the corresponding DFT functionals, are computed from HF orbitals, as MP2 and CCSD­(T), whereas GL2 and all ACII are computed from effective EXX orbitals.^105,106^ Similarly, total energies are reported in Table S3.

From Table 1, we can see that both LDAc and GL2 overestimate the correlation energy by about 5 eV. Recall that these are results without core electrons, and thus quite different from the all-electron case: for example, the MP2 correlation is larger than the CCSD­(T)/CBS reference for copper (the opposite is true in the all-electron case). Recall that the GL2 correlation is computed from EXX orbitals, whereas MP2 is computed from HF orbitals: as the energy-gap is smaller in the former, the GL2 correlation is larger. All ACII methods correct the GL2 overestimation, yielding errors in the range 0.9–0.3 eV, much better than the PBE correlation, which in turn is better than the r^2^SCAN one; note that both the PBE and r^2^SCAN  correlation underestimate the correlation for all atoms. The worst is ISI (MAE ≈ 0.9 eV) followed by revISI (MAE ≈ 0.6 eV). genISI2 and genISI2­[DPI] are quite accurate (MAE ≈ 0.4 eV) and better than MP2: they have almost the same accuracy as uegISI and DPI are quite close for finite systems such as atoms.^47^ The best one is genDPI2-ePC, which reproduces the CCSD­(T)/CBS data with very high accuracy (MAE = 0.26 eV).

## Results for Bulk Valence Correlation Energies

In periodic DFT calculations, the absolute value of the total correlation energy is usually not considered, due to error cancellation with the exchange counterpart, and because it depends on many numerical details (e.g., pseudopotentials, cutoffs, basis sets, ...) which usually cancel out when energy differences are computed (i.e., cohesive energies). On the other hand, in this work, we consider ACII approaches for metals based on the exact exchange and exact (CBS) total energies for atoms, thus the value of correlation energy is meaningful as in CCSD­(T) approaches for bulk.^72–77^

The reference bulk (valence-only) correlation energy per unit cell can be estimated as (see section S6.b)­

19 $$E_{c,Ref.}^{bulk}=\left(e_{HF}^{BSSE}\left(L_{R},B\right)-e_{exp}\right)+E_{c,CCSD\left(T\right)/CBS}^{atom}$$

thus requiring the calculation of the BSSE cohesive energy at the HF level of theory (see Table S5) and the atom correlation at the CCSD­(T)/CBS level of theory (see Table 1). For the experimental cohesive energies we used the values in ref 107.

However, note that the value of $E_{c,Ref.}^{bulk}$ depends on the ECP used. In this work, for each species the same ECP is used for bulk and atomic calculations, since the CRYSTAL23 code can describe both of them with the same setup, and thus our $E_{c,Ref.}^{bulk}$ reference can be used to estimate the quality of the various computational methods for the calculation of the correlation energies in the bulk.

Another implicit assumption of eq 19, is that *e*_exp_ does not have any core contribution. This is certainly valid for the K, Cu, Ag, and Au systems, where there are many valence electrons and the core states (not included in the calculations) are very deep in energy. However, for Li and Na, which are treated here with just one electron in the valence, this assumption must be validated. To estimate the core contribution to the cohesive energy (*e*_core_) of Li and Na, we performed all-electron and ECP calculations with the PBE functional with a very large basis set (see Table S8). We found a core contribution of about 0.10 eV for both Li and Na. This is quite small in absolute value, but it is about 10% of the bulk correlation energy, which thus cannot be neglected. We also verified the core contribution for Al (with only three electrons in the valence): we found 5 · 10^–3^ eV. Thus, in eq 19 we reduce *e*_exp_ by the above *e*_core_ for Li and Na.

The accuracy of eq 19 depends directly on the accuracy of the HF basis set for the HF cohesive energies. We verified this in two different ways. (i) For all systems we compare the $E_{HF}^{ghosts}$ with the CBS one for the isolated atom $E_{HF}^{CBS}$ . Results differ on the average by 0.06 eV, see Table S7. (ii) For all systems we added basis functions with higher angular momentum. The largest deviation in the HF total energy is for Au and it is about 0.05 eV (see Section S7).

Our reference bulk correlation energies, which can be trusted with an error less than 0.1 eV, are reported for all the systems in the last column of Table 2. We also report reference values for Li, Al, and K from literature. Despite the fact that those values are computed with different methods and pseudopotentials, we found very good agreement with our reference values.

**Table 2:**
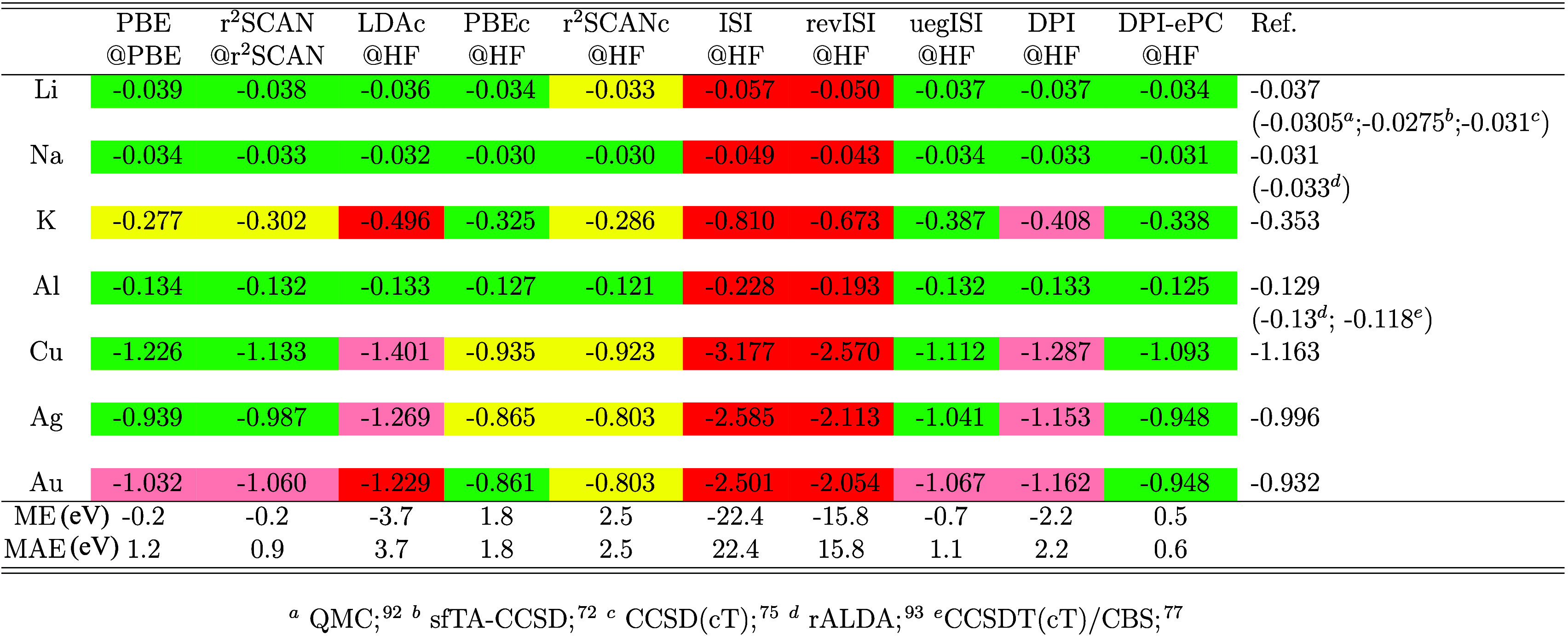
Bulk Correlation Energies Per Atom in a.u. for All Systems and Methods in the DGBO-MTZ Basis Set, and Reference Value from Eq 19 [Table-fn tbl2-fn1]

f Cell colors represents relative deviations, *E* = (*E*_*c*,meth_ – *E*_*c*,*ref*_)/*E_c,ref_*, from reference: red (*E* < 30%), pink (−30% < *E* < −10%), green (−10% < *E* < +10%), yellow (+10% < *E* < +30%), cyan (*E* > +30%). The last two lines report the ME and the MAE in eV.

Table 2 also reports the bulk correlation energies for all the methods investigated in this work. The LDA correlation usually overestimates the true correlation (ME = −3.7 eV), whereas the PBE correlation underestimates it (ME = 1.8 eV) but has a lower MAE. In Table 2 we also report two conventional DFT functionals, using self-consistent orbitals. In these cases, the correlation is computed as $E_{DFT@DFT}^{tot}$ $-E_{HF@HF}^{tot}\;,$ which thus also includes exchange-correlation error compensation and density-driven effects. The accuracy of PBE@PBE increases (MAE = 1.2 eV) as compared to PBEc@HF. The r^2^SCAN  correlation alone is worse than the PBE one, but it is slightly better when it is computed self-consistently (and together with the r^2^SCAN  exchange). ISI and revISI largely overestimate the correlation (with a ME ranging between −22 and −16 eV), as these functionals do not recover the UEG limit. On the other hand, uegISI and DPI have very high accuracy, with a MAE of 1.1 and 2.2 eV, respectively.

The best results are obtained with DPI-ePC, which has a MAE of only 0.6 eV. This result is quite impressive, considering that the DPI-ePC (as all other ACII functionals in the metallic limit) is completely parameter-free, it is based on the Hartree–Fock ground state (which is certainly not the best one to describe metals), and it does not use any information on real solids, but only information from the strong interaction regime.

The very good bulk correlation energies can be explained by investigating the gradient contribution to the bulk correlation. We consider the gradient contribution to the ACII correlation energy, defined as

20 $$\Delta E_{c,\mathrm{ACII}‐\mathrm{GGA}}=G\left(E_{x}^{HF},W_{\infty },W_{\infty }^{\prime }\right)-E_{c,\mathrm{ACII}‐\mathrm{LDA}}$$

with

21 $$E_{c,\mathrm{ACII}‐\mathrm{LDA}}=G\left(E_{x}^{HF},W_{\infty }^{LDA},W_{\infty }^{\prime LDA}\right)$$

i.e., where the strong interaction ingredients $\left(W_{\infty }^{LDA},W_{\infty }^{\prime LDA}\right)$ are computed without any gradient corrections.

In Figure 1a) we report *E*_*c*,ACII‑LDA_/*N* where *N* is the number of valence electrons.

**Figure 1:**
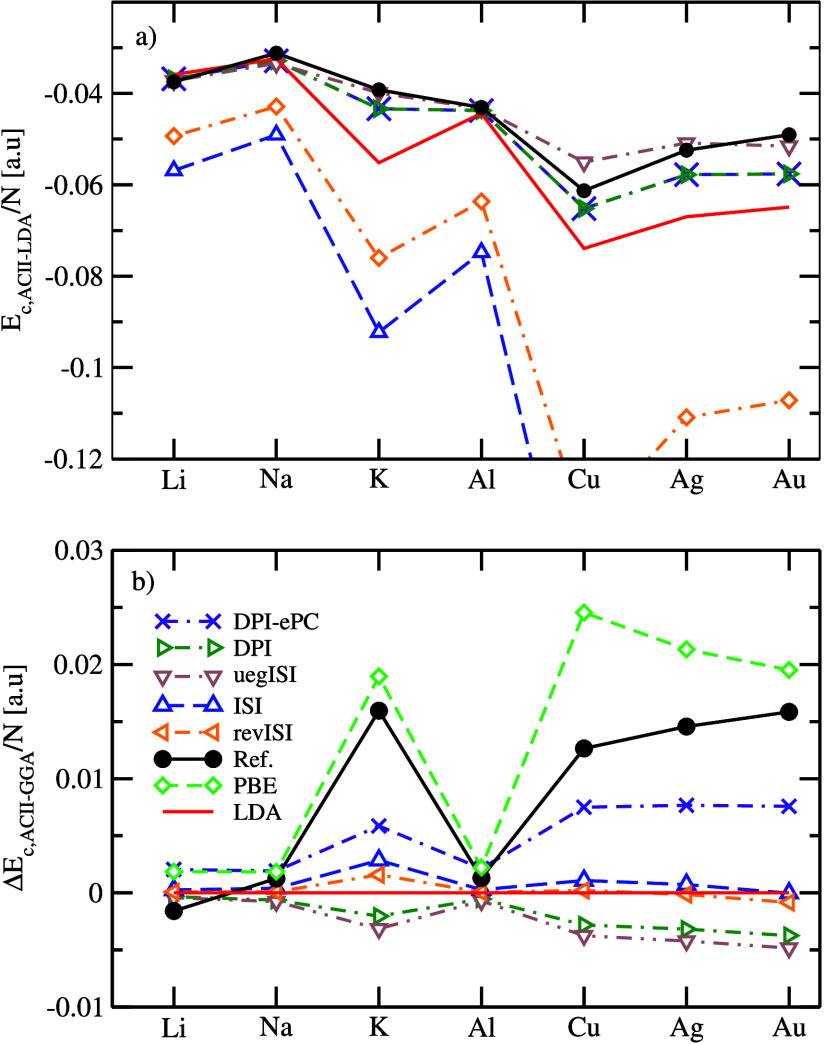
a) *E*_*c*,ACII‑LDA_ divided by the number of valence electrons, for different systems and methods (see text for definition), and b) Δ*E*_*c*,ACII‑GGA_ divided by the number of valence electrons, for different systems and methods (see text for definition).

As previously discussed, ISI and revISI largely overestimate the LDA correlation, which in turn overestimates the exact one. Results for DPI and DPI-ePC, which coincide because hPC and ePC have the same LDA limit, are quite close to the LDA results. On the other hand, uegISI is closer to the exact reference than to LDA.

In Figure 1b) we report Δ*E*_*c*,ACII‑GGA_/*N*. We see that the gradient corrections to ISI and revISI are almost negligible. On the other hand, uegISI and DPI, both with the hPC functional, give *negative* contributions to the correlation energy. This is incorrect because the gradient correction should decrease the correlation, as is the case for the PBE functional. We see that DPI-ePC is the only ACII functional with positive gradient corrections. This is one of the most important result of this work, which confirms the importance of recovering the Wigner crystal limit^54^ for bulk solids.

In Section S8  we also report a similar analysis, based on the jellium model, which confirms that DPI-ePC has a positive second-order gradient expansion (GE2) correlation coefficient. Note that in the PBE correlation the positive GE2 correlation coefficient is imposed by construction,^108^ whereas in DPI-ePC it results from the strong-interaction limit.

## Results for Cohesive Energies

The MAE of cohesive energies are summarized in Figure 2. Full results are reported in Table S9. The ACII cohesive energies are computed as described in Section S6.a. HFLDA, HFPBE, and HFr^2^SCAN  indicate post-HF methods in which the DFT correlation is computed at HF orbitals and added to the HF energy.

**Figure 2:**
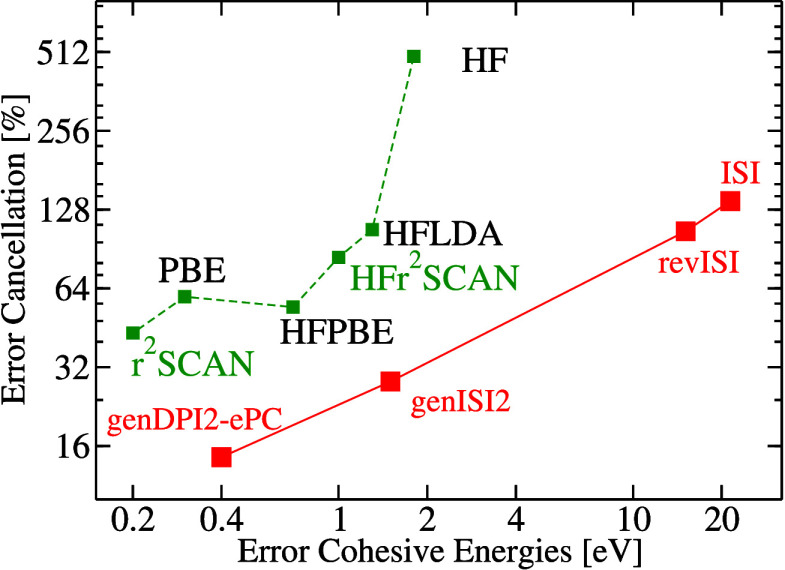
MAE on cohesive energy (eV) vs MAE on Error Cancellation (%), see eq 22, for all methods.

The plot also shows the relation with the averaged *error cancellation*. We define the *error cancellation* of method *M* as

22 $$\Delta_{M}=\mathrm{sign}\left(\Delta_{M}^{bulk}\right)\mathrm{sign}\left(\Delta_{M}^{ghost}\right)\frac{\sqrt{\left|\Delta_{M}^{bulk}\right|\left|\Delta_{M}^{ghost}\right|}}{E_{Ref}^{bulk}-E_{Ref}^{ghost}}$$

where $\Delta_{M}^{ghost}=$ $E_{M}^{ghost}-E_{Ref}^{ghost}$ is the error on total energy for the ghost-atom systems and $\Delta_{M}^{bulk}=$ $E_{M}^{bulk}-E_{Ref}^{bulk}$ is the error on total energy for the bulk. This indicator vanishes if $\Delta_{M}^{ghost}=$ $\Delta_{M}^{bulk}=0$ , i.e., if the method *M* is exact; it also vanishes if either $\Delta_{M}^{ghost}$ or $\Delta_{M}^{bulk}=0$ vanishes, as in this case the error in the cohesive energy of method M is not due to “error cancellation” but because the method overestimates or underestimates the bulk or the ghost-atom system. The typical “error cancellation” is when Δ_*M*_ is large and positive, i.e., when the method M overestimates or underestimates both energies ( $\Delta_{M}^{ghost}$ and $\Delta_{M}^{bulk}$ have the same sign). A less usual case is when Δ_*M*_ is large and negative: in this case $\Delta_{M}^{ghost}$ and $\Delta_{M}^{bulk}$ are large and with opposite sign: in this case the method *M* can be inaccurate as it cannot be corrected by any shift of energies. Full results for all systems are reported in Table S10.

Figure 2 shows that HF underestimates the cohesive energy by about 2 eV/atom, as no correlation is included. With LDA or PBE correlation results are much more accurate (MAE = 0.7 eV for HFPBE) The self-consistent PBE and r^2^SCAN  results are exceptionally good for cohesive energies (MAE = 0.3 eV and MAE = 0.2 eV, respectively), as previously reported in literature.^109–112^ ISI and revISI, which are good for lattice constants, cannot be used at all for cohesive energies, where the errors can be as high as 1000%, in particular for transition metals. The genISI2 functional overestimates much less the cohesive energies, yet there are significant errors for transition metals. Finally, genDPI2-ePC gives very low errors for all systems (but Cu) with a MAE of 0.4 eV, thus comparable with the PBE one.

The key point is, however, that the genDPI2-ePC results are obtained with an almost negligible error cancellation (15%) in contrast to PBE which has an average Δ_PBE_ of 60% even worse than HFPBE. For many systems (K, Cu, Ag, Au) PBE gives very good cohesive energies, but the bulk and the ghost-atom correlation energies are shifted up or down by 1 eV or more (see Tables 1 and 2). Also r^2^SCAN, which is the current state-of-the-art in DFT, has a significant error cancellation $\left(\Delta_{r^{2}\mathrm{SCAN}}=43\%\right)$ .

## Lattice Constants

Table 3 reports the lattice constants for all the systems and methods considered. Our HF lattice constants agree very well with previously reported data^73,113^ with reference or plane-wave basis sets. The same is valid for PBE, which we compare with numerical atomic orbital calculations.^109^ Similar agreement is found for r^2^SCAN (see refs 111, 112 ), which yields the best accuracy. This shows the good accuracy of the optimized basis sets (even if these are optimized at HF and not DFT level).

**Table 3:**
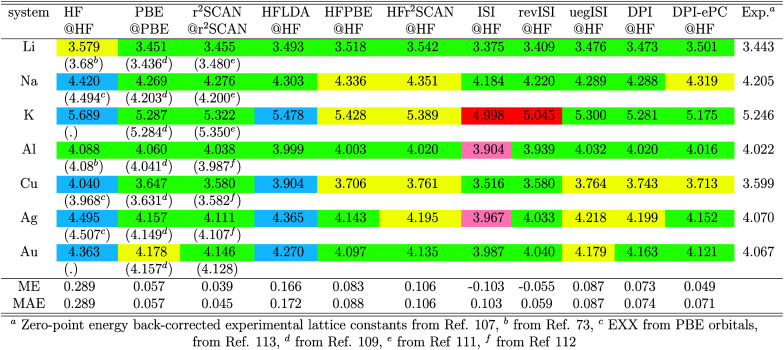
Lattice Constants (in Å) for All Systems and Methods and Statistical Indicators[Table-fn tbl3-fn1]

g Cell colors represents deviations (*E* = *E* ^meth^ – *E* ^Ref.^) from experiments: red (*E* < – 0.2Å), pink (−0.2Å < *E* < – 0.1Å), green (−0.1Å < *E* < + 0.1Å), yellow (0.1Å < *E* < + 0.2Å), cyan (*E* > 0.2Å). Last lines report ME and MAE (in Å).

On average, HF overestimates the lattice constant by more than 0.3 Å. Adding the ISI correlation to HF corrects the overestimation of the lattice constant, but this correction is too large so that ISI underestimates the lattice constants on average by ME = −0.08 Å. The revISI functional has a smaller correlation so that results are improved with a MAE of 0.06 Å, which is the same as PBE. However, as it has been shown above, this is an error cancellation effect as ISI and revISI completely fail for cohesive energies. The uegISI functional, which (partially) recovers the UEG limit, also gives a good description of the lattice constant with ME = MAE = 0.09 Å. With the DPI functional, which fully recovers the UEG limit, the results are further improved (ME = MAE = 0.07 Å.) Note also that DPI yields better lattice constants than the HF plus the LDA correlation approach: these two approaches will be equivalent in the UEG, but in real solids the gradient corrections are also important. Yet, DPI is also better than HFPBE and HFr^2^SCAN.

Finally, with the ePC strong interaction functionals, we obtain a better MAE (0.071 eV) and a better ME (0.049 eV). Among the ACII functionals, DPI-ePC gives very accurate results for Li, K, Al, Ag, and Au (deviation less than about 0.05Å, green entries), whereas a slight overestimation can be seen for Na and Cu.

To analyze the results for lattice constants, the first derivative of the bulk correlation energy vs the lattice constant is important. In fact, near the equilibrium lattice constant, the ACII correlation energies depend (almost) linearly on the lattice constant. We compute the slope (η) of the correlation energy (*E*_*c*_) with respect to the lattice constant (*L*) for a given bulk solid, around the equilibrium lattice constant (*L*_*R*_)­

23 $${\eta =\frac{dE_{c}\left(L\right)}{dL}|}_{L=L_{R}}$$

The “exact” slope can be defined as the one which gives the experimental lattice constant, i.e.

24 $${\eta^{exact}=-\frac{dE^{HF}\left(L\right)}{dL}|}_{L=L_{R}}$$

so that the derivative of the total energy of ACII methods *E*_*c*_ + *E* ^HF^ vanishes.

In Figure 3, we report η for the bulk solids considered. If η < η^exact^ (η > η^exact^), then the lattice constant is overestimated (underestimated). ISI and revISI, which overestimate the correlation energy, always overestimate η (and thus underestimate lattice constants). Nevertheless, η is quite accurate for transition metals. All others ACII underestimate η. HFPBE and genDPI2-ePC are quite accurate for Al and all transition metals, but underestimate Li and Na.

**Figure 3:**
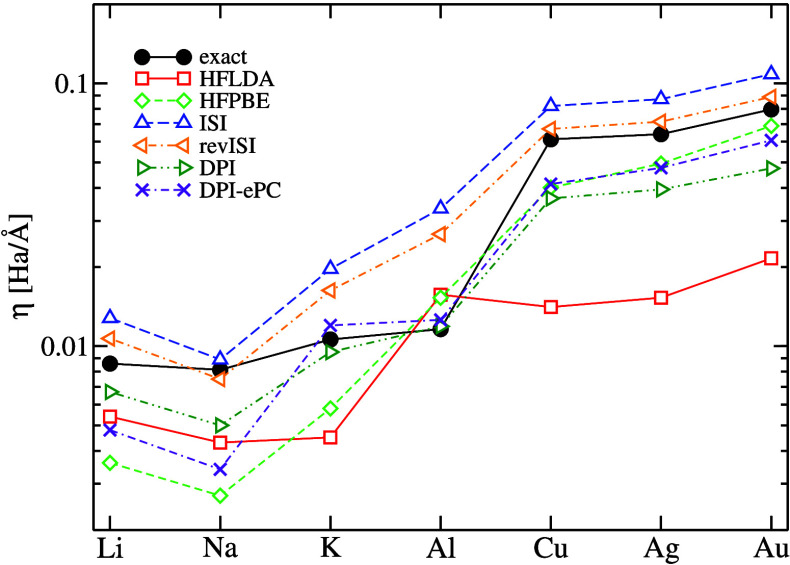
First derivative of the correlation energy versus the lattice constant, for all systems and methods.

Finally, we comment on the bulk moduli (results are reported in Table S14) The description of the bulk modulus is similar to that for lattice constants. For PBE and r^2^SCAN  our bulk moduli agree with literature results as well as with experiment (MARE = 14% and 11%, respectively). Also, our HF bulk moduli agree with the results in the literature. HF yields very incorrect (largely too small) bulk moduli for transition metals. With the ISI functional the bulk modulus becomes too large, whereas revISI is much better, especially for transition metals. For uegISI, DPI, and DPI-ePC the bulk moduli is quite accurate (MARE ≈ 25%), but it is significantly underestimated for transition metals. This behavior is inherited from the very bad HF results, despite the fact that the ACII methods try to correct it by adding the correlation. In those cases we argue that a fully self-consistent ACII calculation can yield better results.

## Summary and Conclusions

In this work we applied ACII functionals to metallic solids. This was a quite challenging task because three different topics had to be faced.

First, the GTO basis sets must be accurate to reproduce the HF ground state. Optimized GTO basis sets with ECP were not available in the literature for all the systems investigated in this work (Li, Na, K, Al, Cu, Ag, Au). Thus, we present here a global optimization scheme without derivatives, named DGBO. This allowed us to obtain the optimized basis set without being stuck in local minima.

Second, as in metallic systems the gap vanishes, and thus the second-order correlation diverges, we used the limiting formula for ACII. We found that ISI and revISI give quite accurate lattice constants, but this is due to error compensation because the bulk correlation energy in this functional is overestimated by one order of magnitude. Thus, improved ACII functionals, which recover the UEG limit (i.e., uegISI and DPI), are required.

The third issue is related to the strong interaction functionals. The conventional PC or hPC strong interaction functionals are based on a second-order gradient expansion that has been fitted to the Helium atom, whereas the exact expansion has even a different sign. In this work, we have shown that the ePC model, which recovers the Wigner crystal limit, is the correct one to describe bulk systems, as it gives a quite correct second order gradient expansion correlation coefficient.

In Figure 4 we report a plot relating the MAE on the lattice constants to the MAE for the bulk correlation energies for all methods. We considered two paths. The green path (green dashed line) indicates the (known) path from HF to r^2^SCAN. As shown in the figure, HF completely misses the correlation energy and overestimates lattice constants. Adding the LDA correlation improves the lattice constants, but the correlation is still overestimated. The PBE correlation is well suited for metallic bulk systems, and indeed HFPBE is better than HFr^2^SCAN and gives quite correct lattice constants and bulk correlation energies. The final step is to perform a self-consistent calculation, improving the ground state orbitals and using the corresponding DFT exchange. PBE (r^2^SCAN) gives very accurate lattice constants, but bulk correlation energies are incorrect by 1.2 eV (0.9 eV).

**Figure 4:**
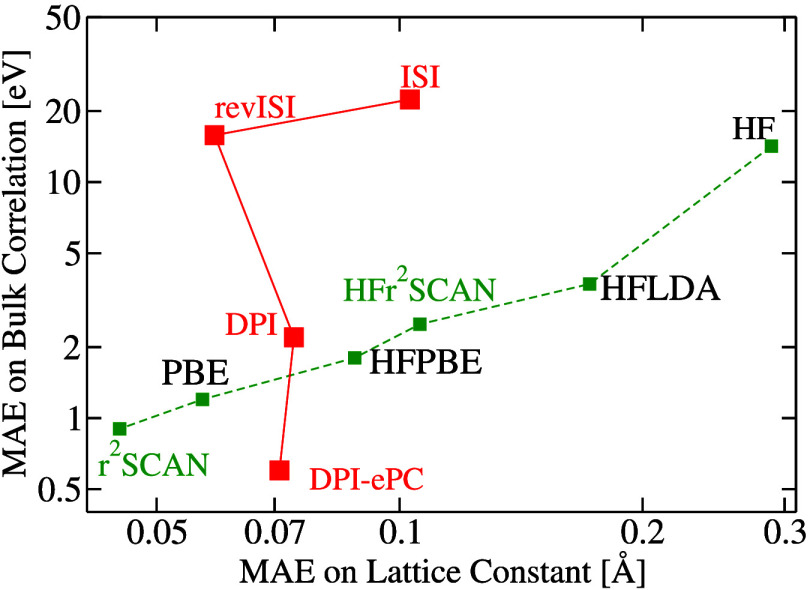
Relation between the MAE on lattice constant and MAE on bulk correlation for all functionals.

The red path (red solid line) shows that ISI gives incorrect lattice constants and correlation energies, while revISI strongly improves, but only for the lattice constants. The UEG limit is very important for ACII methods: in fact, DPI gives quite correct lattice constants and correlation energies. DPI will be equivalent to HFLDA in the UEG. However, in real solids, DPI is much better. The second-order gradient-expansion coefficient is, however, incorrect in DPI (defined with the hPC functional). With the recent ePC model, we find that DPI-ePC is much better than HFPBE and comparable to PBE. Considering that DPI-ePC is not self-consistent and it does not use any information/parameters from any real systems (but only from the strong-interaction regime) the comparison with the self-consistent r^2^SCAN (which employs many constraints and norms from different model systems), can be considered very promising.

For finite systems, DPI-ePC is extended to include the GL2 term. The accuracy of genDPI2-ePC, introduced in this work, is not limited to atoms and cohesive energies, as it is an extension of the genISI2 functional which has already broad applicability.^49,54^ A full benchmark of genDPI2-ePC will be published elsewhere and some relevant results are reported in Section S4.a.

Despite the very good accuracy of the genDPI2-ePC method shown in this work, the present implementation can be further improved and different paths can be considered in the future.

i) In this work we considered HF or EXX for all systems, both due to computational simplicity and to have a common orbital reference. The next step is to develop a self-consistent implementation for bulk metals, which can be easily coded, to further check the accuracy of the ACII functionals.
ii) All the recent ACII functionals (genISI2) and strong interaction functional (ePC) have been developed considering all-electron systems. In this work, we found quite good accuracy also for systems with pseudopotentials, but, clearly, all the above functionals can be reoptimized without the core electrons. Moreover, the more recent ccECP pseudopotentials^114^ can be considered.
iii) The new class of Möller-Plesset-AC functionals, which are based directly on the HF density, can be explored. Such functionals do not require any SCF KS calculation, but require different strong interaction ingredients. However, MP-AC depends also on the density at the core of the atom, which is (almost) zero in the case of pseudopotentials. Thus, further investigations are required.
iv) Calculations of semiconductors can be also explored. This is, in fact, another motivation of implementing ACII functionals into a GTO code: in fact CRYSTAL23 includes the MP2 correlation computation for solids through the CRYSCOR code.^115^

## Supplementary Material



## Notes

ACII interpolation formulas; Computational Details for CRYSTAL23; Construction of the DGBO-MTZ basis set; Atomic data and CBS extrapolation; Details of the genDPI2 functional; Additional tables for the bulk; Derivation of cohesive energies and reference bulk correlation energy; Tables of cohesive energies and error cancellation; Convergence of the DGBO-MTZ basis set; Second-order gradient expansion for jellium; Table with bulk moduli; Complete DGBO-MTZ basis set; Band-structure and density of states. (PDF)
