# Supplementary material for: Adiabatic Connection Correlation Functionals in Metallic Solids from Hartree–Fock Gaussian Basis Set Ground State
Source: J Phys Chem Lett. 2026 Jun 10;17(25):6919–30. doi: 10.1021/acs.jpclett.6c00976 (PMC13312454; doi:10.1021/acs.jpclett.6c00976)
Supplement: Supplementary file 1 [file jz6c00976_si_001.pdf]

Supporting Information for “Adiabatic Connection Correlation Functionals in Metallic Solids from Hartree-Fock Gaussian Basis Set Ground State”

Fabio Della Sala,[1,2] Fulvio Sarcinella,[1,2] Lucian A. Constantin,[1] Lorenzo Donà,[3] Eduardo Fabiano,[1,2] Lorenzo Maschio,[3] and Bartolomeo Civalleri[3]

[1]*Institute for Microelectronics and Microsystems (CNR-IMM), Via Monteroni, Campus Unisalento, 73100 Lecce, Italy*

[2]*Center for Biomolecular Nanotechnologies, Istituto Italiano di Tecnologia, Via Barsanti 14, 73010 Arnesano (LE), Italy*

[3]*Department of Chemistry, University of Turin, via Giuria 7, 10125 Torino, Italy*

(Dated: May 18, 2026)

CONTENTS

S1. AC interpolation formulas S1

S2. Computational Details for CRYSTAL23 S2

S3. Construction of the DGBO-MTZ Basis set S3

S4. Atomic data and CBS extrapolation S4
  A. genDPI2-ePC S5

S5. Additional data for Bulk S6

S6. Cohesive Energy S7
  A. ACII cohesive energy in finite basis set S7
  B. Reference bulk correlation energy S8
  C. Finite basis set correction S8
  D. Tables for cohesive energies S9

S7. Convergence of the DGBO-MTZ basis set S10

S8. Second order gradient expansion correlation coefficient S12

S9. Bulk moduli S14

S10. DGBO-MTZ basis set S14

S11. Band-structure and DOS S19

References S23

S1. AC INTERPOLATION FORMULAS

For ISI[1] we have

$$f_c(x) = x/(1+x), \tag{S1}$$
$$F_c(q) = 1 + 2\ln(q+1)/q^2 - 2/q\ . \tag{S2}$$

For revISI[2] we have

$$f_c(x) = x(x+3)/(x+2)^2, \tag{S3}$$
$$F_c(q) = q/(q+2)\ . \tag{S4}$$

For uegISI[3] we have

$$f_c(x) = \frac{(H(x)^3 + 9.5H(x)^2 - 14H(x) - 18)}{H(x)(7.0 + H(x))^2}, \tag{S5}$$

$$F_c(q) = \frac{-2 + H(q)}{7 + H(q)} \quad \text{with} \tag{S6}$$

$$H(x) = \sqrt{4 + 20.25x^2}\ . \tag{S7}$$

For DPI we start from the original definition [4]

$$\epsilon_c = \frac{(0.009229000000\, rs + 0.03109000000) \ln\left(\frac{rs}{1 + rs}\right) + 0.01526000000 - \frac{0.06218000000}{\sqrt{1 + rs}} - \frac{0.010\, rs}{1 + rs}}{0.5961311610 + \frac{0.4038688390}{(rs^2 + 1)^{1/4}} + 0.1712279179\, (rs^2 + 1)^{1/4} - \frac{0.1802109093}{\sqrt{rs^2 + 1}} + 0.008982991388 \sqrt{rs^2 + 1}} \tag{S8}$$

where r_s is the Wigner-Seitz radius. Then we use the fact that $E_x - W_\infty = 0.4418342367/r_s$ and $r_s = q^2/0.58911^2$, see Ref. 3, obtaining:

$$Fc := -\frac{6.521505203 \left((0.02659266462\, q^2 + 0.03109000000) \ln\left(\frac{2.881424273\, q^2}{1 + 2.881424273\, q^2}\right) + 0.01526000000 - \frac{0.06218000000}{\sqrt{1 + 2.881424273\, q^2}} - \frac{0.02881424273\, q^2}{1 + 2.881424273\, q^2} \right) q^2}{0.5961311610 + \frac{0.4038688390}{(8.302605841\, q^4 + 1)^{1/4}} + 0.1712279179\, (8.302605841\, q^4 + 1)^{1/4} - \frac{0.1802109093}{\sqrt{8.302605841\, q^4 + 1}} + 0.008982991388 \sqrt{8.302605841\, q^4 + 1}} \tag{S9}$$

For the $\alpha-$ dependence we use the uniform scaling relation [5] and that $\alpha r_s = x^2/0.58911^2$, obtaining

$$f_c(x) = p_1 + p_2 + p_3 \tag{S10}$$

with

$$p1 := -\frac{13.04301042\, x^2 \left((0.02659266462\, x^2 + 0.03109000000) \ln\left(\frac{2.881424273\, x^2}{2.881424273\, x^2 + 1}\right) + 0.01526000000 - \frac{0.06218000000}{\sqrt{2.881424273\, x^2 + 1}} - \frac{0.02881424273\, x^2}{2.881424273\, x^2 + 1} \right)}{0.5961311610 + \frac{0.4038688390}{(8.302605841\, x^4 + 1)^{1/4}} + 0.1712279179\, (8.302605841\, x^4 + 1)^{1/4} - \frac{0.1802109093}{\sqrt{8.302605841\, x^4 + 1}} + 0.008982991388 \sqrt{8.302605841\, x^4 + 1}}$$

$$p2 := -\left(18.79122340\, x^4 \left(0.009229000000 \ln\left(\frac{2.881424273\, x^2}{2.881424273\, x^2 + 1}\right) + \frac{0.3470505921\, (0.02659266462\, x^2 + 0.03109000000) \left(\frac{1}{2.881424273\, x^2 + 1} - \frac{2.881424273\, x^2}{(2.881424273\, x^2 + 1)^2}\right) (2.881424273\, x^2 + 1)}{x^2} + \frac{0.03109000000}{(2.881424273\, x^2 + 1)^{3/2}} - \frac{0.010}{2.881424273\, x^2 + 1} + \frac{0.02881424273\, x^2}{(2.881424273\, x^2 + 1)^2}\right)\right) \Big/ \left(0.5961311610 + \frac{0.4038688390}{(8.302605841\, x^4 + 1)^{1/4}} + 0.1712279179\, (8.302605841\, x^4 + 1)^{1/4} - \frac{0.1802109093}{\sqrt{8.302605841\, x^4 + 1}} + 0.008982991388 \sqrt{8.302605841\, x^4 + 1}\right)$$

$$p3 := \left(18.79122340\, x^4 \left((0.02659266462\, x^2 + 0.03109000000) \ln\left(\frac{2.881424273\, x^2}{2.881424273\, x^2 + 1}\right) + 0.01526000000 - \frac{0.06218000000}{\sqrt{2.881424273\, x^2 + 1}} - \frac{0.02881424273\, x^2}{2.881424273\, x^2 + 1}\right) \left(-\frac{0.5818587379\, x^2}{(8.302605841\, x^4 + 1)^{5/4}} + \frac{0.2466901394\, x^2}{(8.302605841\, x^4 + 1)^{3/4}} + \frac{0.5192640883\, x^2}{(8.302605841\, x^4 + 1)^{3/2}} + \frac{0.02588380943\, x^2}{\sqrt{8.302605841\, x^4 + 1}}\right)\right) \Big/ \left(0.5961311610 + \frac{0.4038688390}{(8.302605841\, x^4 + 1)^{1/4}} + 0.1712279179\, (8.302605841\, x^4 + 1)^{1/4} - \frac{0.1802109093}{\sqrt{8.302605841\, x^4 + 1}} + 0.008982991388 \sqrt{8.302605841\, x^4 + 1}\right)^2 \tag{S11}$$

In all the above formula, we have $f_c(x) \to 0$ for small x and $f_c(x) \to 1 - 1/x$ for $x \to \infty$; $F_c(q) \to 0$ for small q and $F_c(q) \to 1 - 2/q$ for $q \to \infty$.

S2. COMPUTATIONAL DETAILS FOR CRYSTAL23

All bulk calculations have been done with the CRYSTAL23 program [6, 7], which is based on Gaussian Type Orbital (GTO) basis sets. We have implemented the hPC[8] and ePC[9] strong-interaction functionals, as post-HF density functionals, in a CRYSTAL23 developing version.

All integrals are selected (FIXINDEX keyword) at the integral reference lattice (a^{IRL}) constant (about 93% of the experimental one, see Tab. S1). In fact, in CRYSTAL23, the computation of the integrals and the Fock matrix depends on numerical tolerances that control the number of integrals to be computed either exactly or approximately

and the truncation of the infinite summation over the direct lattice vectors [7]. Such tolerances refer to atom-centered Gaussian functions, and thus to the geometry of the system. Therefore, in order to avoid numerical noise when changing the lattice constant, the classification and screening of the one- and two-electron integrals are performed at the fixed (a^{IRL}) lattice constant, and then kept fixed for the expanded unit cells.

The truncation criteria ($10^{-\mathrm{ITOL}}$) of Coulomb and exchange infinite sums are set to "ITOL=9 9 9 15 45" for all elements, but for Li and Na (with just one electron in the valence) where we use "ITOL=9 9 9 15 60". For all the calculations the convergence for the SCF process is achieved when the energy difference is 10^{-8} a.u., using shrinking factors of 24 and 48 for the Monkhorst-Pack net and the Gilat nets, respectively.

ACII calculations can be done with the *acmxc* script, which has an interface with CRYSTAL23 and it is freely available [10]. For the optimization of lattice constants we use the *cryslatt* script, which is also freely available [11].

We considered Li, Na, K, Al, Cu, Ag, and Au. For all systems we use the the quasi relativistic effective core potential (ECP) [12–15], as reported in Tab. S1. All ACM calculations are based on the HF density. For comparison, we also performed self-consistent PBE[16] and r^{2}SCAN[17] calculations, considering that PBE and r^{2}SCAN are among the best functionals for the properties of the metallic solids investigated in this work [18–22].

S3. CONSTRUCTION OF THE DGBO-MTZ BASIS SET

The DGBO-MTZ basis set construction is reported in Tab. S1 and it is described in the following.

For Li, Na, Al, Ag and Au we start from the def2-TZVP basis sets [23] as obtained from the Basis Set Exchange repository [24]. Note that the default def2-TZVP for Ag and Au already considered ECP28 and ECP60, respectively, whereas Li, Na and Al are all-electron basis sets, and thus we removed the deepest contraction corresponding to core electrons. For Li we added one d-type function: we found that this decreases the HF energy more than 10^{-4}, which is relevant for accuracy.

For K and Cu we considered the Stuttgart basis sets [24] which consider the ECP10 pseudopotential; for K we also added three d-type uncontracted shells (as in the all-electron def2-TZVP basis set) to the Stuttgart basis set (named Stut.+d).

For Al, Cu, Ag and Au we removed the f-function, which is quite costly, and changed the lattice constant by less than 0.01Å (see Sect. S7 below), which is the overall accuracy.

For Cu we found that the deepest contractions for d-orbital is insufficient for an accurate description. Thus, we also reoptimize all the contractions (we used contraction of five exponents instead of the original four).

Then we applied some modifications required in the solid-state:

We removed some diffuse functions, which are critical in the solid-state, where exponents usually cannot be smaller than about 0.06 a.u: for Li, Na, K, Cu, and Au, we removed the most diffuse s-function, for Li, Na, K, and Ag, we removed the most-diffuse p-function.

For Na we also removed the two tightest d-functions, which we found contributing less than 10^{-5} a.u. to the total energy.

For Al, we replace contracted d-type shell, with one uncontracted.

Then, for the exponent optimization, we optimized all exponents of uncontracted (which are then empty or partially occupied) basis functions.

TABLE S1. From left to right: atom; experimental lattice constant; IRL; Space Group (SG); pseudopotential; starting basis set; definition of the s, p, and d basis set (the format is mL^n, where m is the number of contractions, L is the shell-type (s, p, or d), n, if present, is the formal occupation number), with eventual modifications; total number of optimized exponents indicated with a star (*) in the basis set definition.

	a	a^{IRL}	SG	pseudo	basis	s	p	d	np
Li	3.443	3.25	bcc	ECP2SDF	def2-TZVP+d	~~$6s^2$~~ $(2s^1 \rightarrow 1s^{1*})$ $1s^*$ $1s^*$ ~~$1s^*$~~	$1p^*$ $1p^*$ ~~$1p$~~	$1d^*$	6
Na	4.205	3.9	bcc	ECP10SDF	def2-TZVP	~~$7s^2$~~ ~~$3s^2$~~ $(2s^1 \rightarrow 1s^{1*}\ 1s^*)$ $1s^*$ ~~$1s^*$~~	~~$5p^6$~~ $1p^*$ $1p^*$ ~~$1p$~~	~~$1d$~~ ~~$1d$~~ $1d^*$	6
K	5.246	4.8	bcc	ECP10MWB	Stut.+d	$3s^2$ $1s^{1*}$ $1s^*$ $1s^*$ ~~$1s$~~	$3p^6$ $1p^*$ $1p^*$ ~~$1p$~~	$1d^*$ $1d^*$ $1d^*$	8
Al	4.022	3.7	fcc	ECP10MWB	def2-TZVP	~~$7s^2$~~ ~~$3s^2$~~ $(2s^2 \rightarrow 1s^{2*}\ 1s^*)$ $1s^*$ $1s^*$	~~$5p^6$~~ $1p^{1*}$ $1p^*$ $1p^*$ $1p^*$	$(2d \rightarrow 1d^*)$ $1d^*$	10
Cu	3.599	3.4	fcc	ECP10MWB	Stut.	$3s^2$ $1s^{1*}$ $1s^*$ $1s^*$ $1s^*$ ~~$1s$~~	$(2p\ 2p)^6$ $1p^*$ $1p^*$ $1p^*$	$5d^{10}$ $1d^*$ $1d^*$	9
Ag	4.070	3.8	fcc	ECP28MWB	def-TZVP	$2s^2$ $1s^{1*}$ $1s^*$ $1s^*$ $1s^*$ $1s^*$	$4p^6$ $1p^*$ $1p^*$ ~~$1p$~~	$4d^{10}$ $1d^*$ $1d^*$	9
Au	4.067	3.8	fcc	ECP60MWB	def-TZVP	$3s^2$ $1s^{1*}$ $1s^*$ $1s^*$ $1s^*$ ~~$1s$~~	$4p^6$ $1p^*$ $1p^*$ $1p^*$	$4d^{10}$ $1d^*$ $1d^*$	9

S4. ATOMIC DATA AND CBS EXTRAPOLATION

We employed very large uncontracted basis sets constructed as follows: for each angular momentum $l = s, p, d, f, g, h, i$, we selected the largest exponent of the corresponding shell in the starting basis set [25, 26] (reported in Tab. S2); in some cases one or two tightest s-exponent have been removed to avoid spurious peaks in the density at the core. All other exponents were then generated using a geometric progression with a ratio of 1.7, until the full-width-at-half-maximum of the most diffuse Gaussian reached 30 a.u. The final number of exponents for each angular momentum is reported in Tab. S2.

Then a CBS extrapolation was performed using the three-point formula

$$E(l_{\max}) = E_{\rm CBS} + A\, l_{\max}^{-\alpha}, \tag{S12}$$

with the fit carried out for $l_{\max} = 4, 5, 6$, i.e. the basis functions in Tab. S2 up to g, h, i, respectively. The extrapolation was applied to both MP2 and CCSD(T) correlation energies. In the case of MP2, the same-spin (SS) and opposite-spin (OS) correlation contributions were extrapolated separately, as they are known to exhibit different convergence rates toward the CBS limit [27]. All other quantities, that depend solely on ground-state orbitals, i.e. the Hartree–Fock energy, exchange energy, and strong-correlation functionals, are independent of $l_{\max}$ for $l_{\max} > 3$ because these are spherical systems whose occupied orbitals have contributions only from basis functions up to d-type.

TABLE S2. From left to right: atom, starting basis set, max s-exponent (removed s-exponents), CBS basis set, HF total energy, and PBE self-consistent total energy, for all systems. Note that for Li and Na, the starting basis set do not include i functions: in this case exponents for the i functions are the same as the h functions. Total energies in a.u. from TURBOMOLE.

atom	starting basis set	max s expon. (rem.)	basis set	HF	PBE
Li	ccecp-aug-cc-pV5Z	3.593690000 (2)	$16s$ $16p$ $11d$ $11f$ $9g$ $9h$ $(9i)$	-0.19603218370	-0.20066943900
Na	ccecp-aug-cc-pV5Z	9.660600000 (1)	$18s$ $16p$ $9d$ $9f$ $9g$ $7h$ $(7i)$	-0.18212693438	-0.18775885118
K	ccecp-aug-cc-pCV6Z	33.19060000 (0)	$21s$ $18p$ $18d$ $14f$ $13g$ $12h$ $11i$	-28.04134653370	-28.28888414379
Al	ccecp-aug-cc-pV6Z	8.257940000 (0)	$21s$ $18p$ $18d$ $14f$ $13g$ $12h$ $11i$	-1.88315538746	-1.94314070746
Cu	ccecp-aug-cc-pCV5Z	104.4710000 (0)	$23s$ $22p$ $22d$ $16f$ $15g$ $14h$ $14i$	-196.22185388533	-197.35848028409
Ag	ccecp-aug-cc-pCV5Z	57.33890000 (2)	$22s$ $20p$ $19d$ $15f$ $13g$ $13h$ $13i$	-146.11826534613	-146.98676642234
Au	ccecp-aug-cc-pCV5Z	233.3410000 (0)	$24s$ $20p$ $19d$ $13f$ $13g$ $13h$ $12i$	-134.79019403656	-135.74733183945

TABLE S3. Total energies in a.u. for all systems and methods. MAE and ME in eV. PBE, LDA and r^{2}SCAN are computed self-consistently. MP2 uses HF orbitals. GL2 and all ACM use EXX(LHF) orbitals and total energies. Cell colors represent errors E with respect to CCSD(T): red ($E < -0.05$), pink ($-0.05 < E < -0.02$), green ($-0.02 < E < +0.02$), yellow ($+0.02 < E < +0.05$), cyan ($E > +0.05$).

sys	LDA @LDA	PBE @PBE	r^{2}SCAN @r^{2}SCAN	MP2 @HF	GL2 @EXX	ISI @EXX	revISI @EXX	gISI2 @EXX	gISI2[DPI] @EXX	gDPI2-ePC @EXX	CCSD(T) @HF
Li	-0.19413	-0.20067	-0.19668	-0.19603	-0.19603	-0.19603	-0.19603	-0.19603	-0.19603	-0.19603	-0.19603
Na	-0.18145	-0.18776	-0.18332	-0.18213	-0.18213	-0.18213	-0.18213	-0.18213	-0.18213	-0.18213	-0.18213
K	-28.19178	-28.28888	-28.31541	-28.33714	-28.47444	-28.36050	-28.35414	-28.32846	-28.33161	-28.36517	-28.36155
Al	-1.92359	-1.94314	-1.94014	-1.92479	-1.96430	-1.93499	-1.93536	-1.93424	-1.93336	-1.94550	-1.93881
Cu	-197.00649	-197.35848	-197.23768	-197.36973	-197.70743	-197.38786	-197.35452	-197.28622	-197.30921	-197.24626	-197.27726
Ag	-146.71315	-146.98677	-147.01962	-147.04289	-147.32680	-147.05825	-147.03720	-146.97443	-146.98977	-146.98369	-147.02223
Au	-135.50317	-135.74733	-135.76010	-135.63292	-135.89278	-135.65567	-135.64079	-135.58781	-135.60002	-135.59934	-135.60805
ME(eV)	3.39	-0.49	-0.26	-0.39	-4.50	-0.74	-0.44	0.38	0.17	0.26	
MAE(eV)	3.39	1.33	0.95	0.69	4.50	0.77	0.53	0.45	0.42	0.34	

A. genDPI2-ePC

The expression of the genDPI2-ePC functional (where 2 indicates two parameters) is

$$W_{xc,\alpha}^{\text{genDPI2}} = W_{xc,\alpha}^{\text{DPI}} + W_{xc,\alpha}^{a_1} + W_{xc,\alpha}^{a_2}, \tag{S13}$$

$$W_{xc,\alpha}^{a_1} = \frac{W_0'[n]\alpha}{(1 + r_1[n]\alpha E_{\text{GL2}}/W_0[n])^3} \leq 0, \tag{S14}$$

$$W_{xc,\alpha}^{a_2} = \frac{-W_{xc,\alpha}^{\text{DPI}}[n] + W_0[n]}{1 + r_2[n](\alpha E_{\text{GL2}}/W_0[n])^2} \geq 0, \tag{S15}$$

$$r_1[n] = l_1(W_\infty[n]/W_0[n])^3 \tag{S16}$$

$$r_2[n] = l_2(W_\infty[n]/W_0[n])^2 \tag{S17}$$

The main functional difference with respect to genISI2 is the denominator of $W_{xc,\alpha}^{a_2}$, which does not have any linear term in α: this is required as $W_{xc,\alpha}^{\text{DPI}}$ has an infinite derivative at $\alpha = 0$. The different definition of $r_1[n]$ and $r_2[n]$ is instead related to the parametrization on systems with pseudopotentials, whereas genISI2 was parametrized with all-electron basis sets.

We fit the l_1 and l_2 parameters of the genDPI2-ePC functional, towards the CCSD(T)/CBS correlation energies in Tab. I in the manuscript. As those are all open-shell systems, we consider additional 5 closed-shell systems, namely the conventional noble gas atoms (Ne, Ar, Kr) and the transition metals with filled d-shell (Pd, Zn), computed with the same CBS computational scheme. The fitting is done minimizing the RMS error of those 10 systems (Li and Na, with one electron only are clearly excluded, as the correlation is null). As there are two parameters in the $W_{xc,\alpha}^{\text{genDPI2}}$ expression and the total energy is the integral over $\alpha = 0..1$, there is quite a large region of parameters with similar accuracy. In order to remove some more oscillating solutions, we enforce that $\partial W_{xc,\alpha}^{\text{genDPI2}}/\partial\alpha \approx \partial W_{xc,\alpha}^{DPI2}/\partial\alpha$ at the $\alpha = \alpha_m$ which is defined by $W_{xc,\alpha}^{\text{DPI}} = W_{xc,\alpha}^{EGL2} = \alpha W_0'[n]$. The coefficients and the resulting RMS for the 10 systems and the MAE for the 5 metals are reported in Tab. S4. The improvement in the MAE is not very large, meaning that DPI-ePC is also quite accurate also for the considered metal atom.

TABLE S4. Opimized Parameters (l_1 and l_2) for genDPI2, RMS for the 10 atoms benchmark, and MAE for the 5 metals. All values are in a.u..

sys	l_1	l_2	RMS(all)	MAE(K,Al,Cu,Ag,Au)
DPI-ePC	∞	∞	0.027182	0.01409
genDPI2-ePC	5.25	17.3	0.009128	0.00948

A detailed analysis of the genDPI2 functional with further optimizations will be discussed elsewhere. Here, we only report the results of the two benchmarks as reported in Ref. [9]. the AE6 and G21IP test (which include organic systems not considered at all in the above parametrization). We obtained 7.6 kcal/mol and 2.9 kcal/mol. These results are in line or even better than the genISI2-hPC data[9], 13.8 kcal/mol and 2.4 kcal/mol, respectively.

S5. ADDITIONAL DATA FOR BULK

TABLE S5. HF self-consistent total energy for the bulk and isolated atom with ghost functions in a.u. and their difference (ghost−bulk, in eV), at experimental lattice constant. All values in a.u. For the Aluminum isolated atom, CRYSTAL has a problem with the spin, thus result is from TURBOMOLE.

sys	$E_{\mathrm{HF}}^{\mathrm{bulk}}$ [a.u.]	$E_{\mathrm{HF}}^{\mathrm{ghost}}$ [a.u.]	$e_{\mathrm{HF}}^{\mathrm{BSSE}}(L_R)$ [eV]
Li	-0.2158693	-0.1959745	0.54137
Na	-0.1880371	-0.1820294	0.16348
K	-28.0398602	-28.0379068	0.05315
Al	-1.9334071	-1.8810429*	1.42491
Cu	-196.2360472	-196.2145383	0.58529
Ag	-146.1331975	-146.1165407	0.45326
Au	-134.8153211	-134.7884339	0.73164

TABLE S6. PBE self-consistent energy for the bulk and isolated atom with ghost functions in a.u. and their difference (ghost−bulk, in eV), at experimental lattice constant. For the Aluminum isolated atom, CRYSTAL has a problem with the spin, thus result is from TURBOMOLE.

sys	$E_{\mathrm{PBE}}^{\mathrm{bulk}}$ [a.u.]	$E_{\mathrm{PBE}}^{\mathrm{bulk}}$ [a.u.]	$e_{\mathrm{PBE}}^{\mathrm{BSSE}}(L_R)$ [a.u.]
Li	-0.2549076	-0.2006243	1.47712
Na	-0.2227016	-0.1876557	0.95365
K1	-28.3174371	-28.2850454	0.88142
Al	-2.0681528	-1.9413688	3.44997
Cu	-197.4244756	-197.3010320	3.35907
Ag	-147.0757654	-146.9841001	2.49434
Au	-135.8520132	-135.7455336	2.89746

TABLE S7. Difference in the atomic HF energy between the DGBO basis set with ghosts functions (Tab. S5) with the CBS one (Tab. S4). Last line is the MAE in eV.

Li	0.000057
Na	0.000097
K1	0.003439
Al	0.002112
Cu	0.007315
Ag	0.001724
Au	0.001760
MAE(eV)	0.06

TABLE S8. PBE cohesive energies at (experimental lattice constant) in eV using all-electron and ECP, their difference (e_{core}) and the corresponding basis set. We used an almost complete basis set, i.e. geometric series with progression factor 2.5. The exponents for the ECP basis set are the same as the all-electron case, but some of the tightest ones are removed.

system	$e_{\mathrm{PBE,all}}^{\mathrm{BSSE}}$	$e_{\mathrm{PBE,ECP}}^{\mathrm{BSSE}}$	e_{core}	basis set(All/ECP)
Li	1.6089499	1.5007302	0.10822	13s 5p 1d / 5s 5s 1d
Na	1.0802135	0.9728972	0.10731	15s 9p 4d / 6s 6s 2d
Al	3.6150067	3.6096678	0.00533	14s 10p 3d / 5s 4s 3d

S6. COHESIVE ENERGY

For a given basis set B, a given lattice constant L, the BSSE cohesive energy of method M is:

$$e_M^{\text{BSSE}}(L,B) = -E_M^{\text{bulk}}(L,B) + E_M^{\text{ghost}}(L,B) > 0 \tag{S18}$$

where E are total energies and *ghost* indicates a system with a single atom surrounded by shells of ghost atoms at the lattice position: clearly, $E_M^{\text{ghost}}(L,B)$ depends on L, as the position of the basis set functions of the ghost atoms.

In the CBS limit (C), no BSSE is required, and we have

$$\begin{aligned} e_M^{\text{BSSE}}(L,B) = e_M(L,C) = \\ -E_M^{\text{bulk}}(L,C) + E_M^{\text{atom}}(C) > 0 \end{aligned} \tag{S19}$$

as with a complete basis set the isolated atom or with ghost atoms will be same,

For comparison with experiments the cohesive energies are usually compute at the lattice constant optimized for the method met ($L = L_M$).

$$e_M(L_M,C) = -E_M^{\text{bulk}}(L_M,C) + E_M^{\text{atom}}(C) > 0 \tag{S20}$$

The exact reference (R), i.e. from experiments, cohesive energy is

$$e_R(L_R,C) = -E_R^{\text{bulk}}(L_R,C) + E_R^{\text{atom}}(C) > 0 \tag{S21}$$

A. ACII cohesive energy in finite basis set

Using Eq. (4) and Eq. (12) in the manuscript, we have

$$\begin{aligned} e_{\text{ACII}}^{\text{BSSE}}(L,B) = -E_{\text{HF}}^{\text{bulk}}(L,B) - \mathcal{G}(\underline{W}_{\text{HF}}^{\text{bulk}}(L,B)) \\ +E_{\text{EXX}}^{\text{ghost}}(L,B) + \mathcal{F}(\underline{W}_{\text{EXX}}^{\text{ghost}}(L,B), E_{\text{GL2@EXX}}^{\text{ghost}}(L,B)) \end{aligned} \tag{S22}$$

where $E_{\text{HF}}^{\text{bulk}}$ in the HF total SCF energy of the bulk, $E_{\text{EXX}}^{\text{ghost}}$ is the EXX total SCF energy of atom+ghosts system, and $\underline{W}$ collects the three ACII ingredients, i.e. E_x, W_∞, W'_∞ and the subscript indicates which orbitals (and density) are used.

Now we use the fact that for ground-state orbitals and density, HF and EXX are almost equivalent, and thus

$$\begin{aligned} e_{\text{ACII}}^{\text{BSSE}}(L,B) \approx -E_{\text{HF}}^{\text{bulk}}(L,B) - \mathcal{G}(\underline{W}_{\text{HF}}^{\text{bulk}}(L,B)) \\ +E_{\text{HF}}^{\text{ghost}}(L,B) + \mathcal{F}(\underline{W}_{\text{HF}}^{\text{ghost}}(L,B), E_{\text{GL2@EXX}}^{\text{ghost}}(L,B)) \end{aligned} \tag{S23}$$

It is worth to note that GL2 still needs to be computed on EXX orbitals, not on HF orbitals.

In a complete basis set (C) we have from Eq. (S19)

$$\begin{aligned} e_{\text{ACII}}(L;C) \approx -E_{\text{HF}}^{\text{bulk}}(L,C) - \mathcal{G}(\underline{W}_{\text{HF}}^{\text{bulk}}(L,C)) \\ +E_{\text{HF}}^{\text{ghost}}(C) + \mathcal{F}(\underline{W}_{\text{HF}}^{\text{ghost}}(C), E_{\text{GL2@EXX}}^{\text{ghost}}(C)) \end{aligned} \tag{S24}$$

as the ghost-atom system will not be dependent on L (because the basis set is complete).

Then we consider the following approximation for the HF energy of the bulk

$$E_{\text{HF}}^{\text{bulk}}(L,C) \approx E_{\text{HF}}^{\text{bulk}}(L,B) + (E_{\text{HF}}^{\text{ghost}}(C) - E_{\text{HF}}^{\text{ghost}}(L,B)) \tag{S25}$$

i.e. we add a basis set correction obtained from the ghost atom system. This is, of course, valid only if the basis B is already very good to describe the atoms interaction in the bulk, but can be limited in the description of the inner region of the atom (where this correction works).

Thus we have:

$$
\begin{aligned}
&e_{\mathrm{ACII}}(L;C) \approx -E_{\mathrm{HF}}^{\mathrm{bulk}}(L,B) - \mathcal{G}(\underline{W}_{\mathrm{HF}}^{\mathrm{bulk}}(L,C)) \\
&+E_{\mathrm{HF}}^{\mathrm{ghost}}(L,B) + \mathcal{F}(\underline{W}_{\mathrm{HF}}^{\mathrm{ghost}}(C), E_{\mathrm{GL2@EXX}}^{\mathrm{ghost}}(C))
\end{aligned} \tag{S26}
$$

Then we consider that all the $\underline{W}$ quantities depend on occupied orbitals only (or on the density) and thus if the basis set B is already quite accurate, these values computed can be assumed to be close to a complete basis:

$$
\begin{aligned}
&e_{\mathrm{ACII}}(L;C) \approx -E_{\mathrm{HF}}^{\mathrm{bulk}}(L,B) - \mathcal{G}(\underline{W}_{\mathrm{HF}}^{\mathrm{bulk}}(L,B)) \\
&+E_{\mathrm{HF}}^{\mathrm{ghost}}(L,B) + \mathcal{F}(\underline{W}_{\mathrm{HF}}^{\mathrm{ghost}}(L,B), E_{\mathrm{GL2@EXX}}^{\mathrm{ghost}}(C)) \\
&= -E_{\mathrm{ACII}}^{\mathrm{bulk}}(L,B) + E_{\mathrm{ACII}}^{\mathrm{ghost}}(L,B/C)
\end{aligned} \tag{S27}
$$

which coincides with Eq. (S18), but for the term with the ghost atoms in which the GL2 correlation needs to be computed from the isolated atom in a complete basis set.

B. Reference bulk correlation energy

Eq. (S21) with an reference (Ref.) level of theory (e.g. CCSD(T)) becomes

$$
\begin{aligned}
e_{\mathrm{exp}} = e_R(L_R,C) = &-E_{\mathrm{HF}}^{\mathrm{bulk}}(L_R,C) - E_{c,Ref.}^{\mathrm{bulk}}(L_R,C) \\
&+E_{\mathrm{HF}}^{\mathrm{atom}}(C) + E_{c,Ref.}^{\mathrm{atom}}(C)
\end{aligned} \tag{S28}
$$

where e_{exp} is the experimental cohesive energy assumed to be equal to the one obtained from the reference level of theory. Thus, the reference bulk correlation energy is

$$
\begin{aligned}
&E_{c,Ref.}^{\mathrm{bulk}}(L_R,C) = +E_{\mathrm{HF}}^{\mathrm{atom}}(C) + E_{c,Ref.}^{\mathrm{atom}}(C) \\
&-E_{\mathrm{HF}}^{\mathrm{bulk}}(L_R,C) - e_{\mathrm{exp}}
\end{aligned} \tag{S29}
$$

Now we use again Eq. (S25) obtaining:

$$
\begin{aligned}
&E_{c,Ref.}^{\mathrm{bulk}}(L_R,C) = +E_{\mathrm{HF}}^{ghost}(L_R,B) + E_{c,Ref.}^{\mathrm{atom}}(C) \\
&-E_{\mathrm{HF}}^{\mathrm{bulk}}(L_R,B) - e_{\mathrm{exp}} \\
&= (E_{\mathrm{HF}}^{BSSE}(L_R,B) - e_{\mathrm{exp}}) + E_{c,Ref.}^{\mathrm{atom}}(C).
\end{aligned} \tag{S30}
$$

Eq. S30 requires just the calculation of the HF cohesive energy at the experimental lattice constant, with an accurate basis set B and the BSSE correction (see Tab. S5) and a reference calculation of the free atom (e.g. CCSD(T)). In this way a direct calculation of the correlation energy for the bulk with an accurate method (i.e. the calculation of $E_{c,Ref.}^{\mathrm{bulk}}(L_R,C)$) is avoided.

C. Finite basis set correction

We report in Fig. S1, the cohesive energy curve for gold, with the HF method. The energy $E^{\mathrm{ghost}}(L)$ is clearly lower than E^{atom}, as the basis set is larger, and it will only approach it for large L. Despite the energy difference is small, about 10^{-4} a.u., the shift of the predicted lattice constant minimum is quite evident (about 0.02 Å, which is, however, much better than the 0.05 Å shift reported with smaller basis sets [28]). However, HF is the worst case, whereas for other functionals investigated in this work the shift is smaller than 0.01 Å, which can be thus considered the overall accuracy of our basis set.

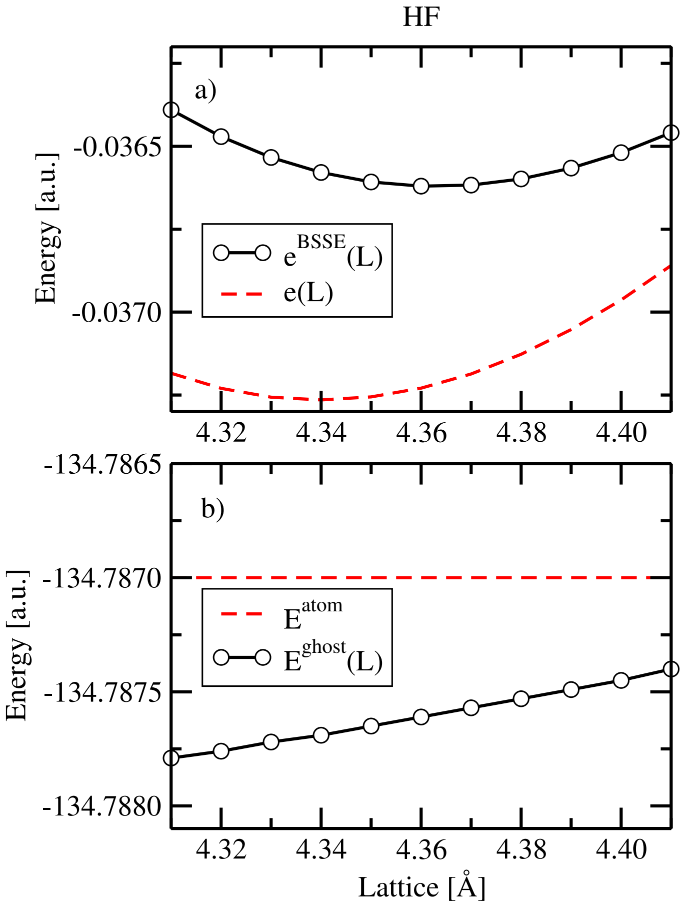

FIG. S1. Cohesive-energy ($e(L)$) and BSSE-corrected cohesive energy ($E^{\mathrm{BSSE}}(L)$) for Au, obtained from Hartree-Fock (HF) (a). Panel b) shows HF atom energy from an isolated atom (E_{atom}) and using ghost-basis functions ($E^{\mathrm{ghost}}(L)$).

D. Tables for cohesive energies

TABLE S9. Cohesive energies in eV/atom. Cell colors represent deviations ($E = E^{\mathrm{meth}} - E^{\mathrm{Ref.}}$) from experiments: red ($E < -2eV$), pink ($-2eV < E < 0.5eV$), green ($-0.5eV < E < +0.5eV$), yellow ($0.5eV < E < 2eV$), cyan ($E > 2eV$).

system	HF @HF	PBE @PBE	r^2SCAN @r^2SCAN	HFLDA @HF	HFPBE @HF	HFr^2SCAN @HF	ISI @EXX	revISI @EXX	genISI2 @EXX	genISI2[DPI] @EXX	genDPI2-ePC @EXX	Ref.[a]
Li	0.56 (0.59[b])	1.48 (1.68[d])	1.56 (1.57[e])	1.14	1.30	1.45	2.09	1.88	1.57	1.55	1.49	1.67
Na	0.19 (0.23[c])	0.96 (1.10[d])	1.05 (1.09[e])	0.68	0.84	1.00	1.49	1.33	1.10	1.08	1.01	1.12
K	0.11 (.)	0.88 (0.88[d])	0.85 (0.85[e])	0.50	0.69	0.75	13.26	9.75	2.75	3.24	0.38	0.94
Al	1.43 (1.39[b])	3.45 (3.47[d])	3.45 (3.60[e])	2.24	3.24	3.31	6.12	5.20	3.62	3.66	3.11	3.43
Cu	0.88 (0.03[c])	3.42 (3.52[d])	3.85 (3.87[f])	1.40	2.09	1.92	54.87	39.41	1.82	5.96	2.33	3.51
Ag	0.75 (0.52[c])	2.52 (2.54[d])	2.87 (2.89[f])	1.23	1.99	1.82	44.83	32.71	5.53	8.14	2.66	2.96
Au	1.00 (.)	2.95 (3.09[d])	3.29 (3.41[f])	1.48	2.58	2.30	44.76	33.16	7.97	10.20	4.31	3.83
ME	-1.8	-0.3	-0.1	-1.3	-0.7	-0.7	21.4	15.1	1.0	2.3	-0.3	
MAE	1.8	0.3	0.2	1.3	0.7	0.7	21.4	15.1	1.5	2.4	0.4	

[a] from Ref. 29, [b] from Ref. 30, [c] EXX from PBE orbitals, from Ref. 31, [d] from Ref. 18, [e] from Ref. 22, [f] from Ref. 21

TABLE S10. Error cancellation Δ (in percent) for all systems and methods. Last line reports the ME and the MAE. Cell colors represent: red ($\Delta < -100\%$), pink ($-100\% < \Delta < -25\%$), green ($-25\% < \Delta < +25\%$), yellow ($25\% < \Delta < +100\%$), cyan ($\Delta > +100\%$).

system	PBE @PBE	r^2SCAN @r^2SCAN	HF @HF	HFLDA @HF	HFPBE @HF	HFr^2SCAN @HF	ISI @EXX	revISI @EXX	genISI2 @EXX	genISI2[DPI] @EXX	genDPI2-ePC @EXX
Li	5.9	2.5	-19.8	-8.9	-9.8	-6.8	14.1	11.1	1.1	-2.0	-5.4
Na	13.8	8.6	-26.7	10.9	-6.3	-4.7	19.9	16.2	8.2	7.1	-2.7
K	224.0	151.4	979.9	427.8	73.0	188.4	-104.5	-156.8	-95.6	-118.3	-16.3
Al	2.4	-0.3	69.0	7.2	-2.6	5.7	-17.4	-13.5	-1.8	-2.6	-4.6
Cu	46.7	34.2	863.8	211.9	157.2	162.4	368.3	258.3	-18.7	49.8	35.1
Ag	42.2	6.0	872.8	282.6	101.4	153.4	231.3	133.5	-42.4	-62.2	36.2
Au	84.4	99.2	620.7	242.8	30.1	67.2	209.9	151.9	-29.4	-4.7	1.3
MAE	59.9	43.2	493.2	170.3	54.3	84.1	137.9	105.9	28.2	35.2	14.5

S7. CONVERGENCE OF THE DGBO-MTZ BASIS SET

We tested the convergence of the DGBO-MTZ basis-set adding basis functions with higher angular momentum (e.g. f and g functions). The added f and/or g exponents have been reoptimized with DGBO (starting from the original def2-TZVP or def2-QZVP exponents), while all the other exponents (e.g. s,p,d) are kept fixed.

Adding more basis functions for the valence orbitals, i.e. s and p functions, is very often not possible, as the exponents are limited both from above (due to the deeper contractions, as in K,Cu,Ag,Au) and from below (as exponents cannot be smaller than about 0.1-0.06). Adding more functions will make the exponents very close to each other and the condition number of the overlap matrix will rapidly increase: thus the HF SCF will fail.

For systems without contractions in the valence (e.g. Li,Na,Al) the optimization procedure (which minimizes the conditioned energy) will move the additional exponent to very high values, in order to minimize the condition number: but such high exponents have almost no impact on total energy as the density vanishes at the core due to pseudopotentials. We tested it for Li and Na. Similar findings have been found in Ref. [32]. Note that calculations with semilocal DFT are easier to converge with larger basis sets, but this is not the case when the full non-local HF exchange is included. The reason for this behaviour is the problem with the long range decay of the HF density matrix[33].

In Tab. S11 we report the data of the original DGBO-MTZ and its extension. In detail: For Li adding 2s1p1d functions to the DGBO-MTZ (so that the full basis set is 5s3p2d) and reoptimizing all exponents has no effect: the HF energy changes by 1meV (see last column of Tab. S11). The optimized s-exponents are: 500, 70, 4.10, 0.53, 0.0631. Comparing with the DGBO-MTZ ones (4.10, 0.53, 0.0640), we see that the additional s-functions are moved to very high exponents, which has no effect on total energy. Also, replacing the 1d function with (reoptimized) 2d1f functions (which are in the def2-QZVP) has no effect.

For Na adding 1s1p1d function (so that the full basis will be 4s3p2d) and reoptizing all exponents, has almost no effect (3 meV shift). The optimized s-exponents are: 260, 3.50, 0.460, 0.0553. Comparing with the DGBO-MTZ ones (3.20, 0.460, 0.590), we see that the additional s-function are moved to a very high exponent, which has no effect on total energy. Replacing the 1d function with (reoptimized) 3d1f functions (which are in the def2-QZVP) has almost no effect.

For K adding one (reoptimized) one f-type function, has no effect.

For Al adding one (reoptimized) f-type function (which is present in the original def2-TZVP) will decrease the energy of 0.027 eV; further addition of a g-type function has no effect.

For Cu adding one (reoptimized) f-type function (which is present in the original def2-TZVP) will decrease the energy of 0.003 eV; further addition of 1f1g functions decreases the energy of only 6 meV.

For Ag adding one (reoptimized) f-type function (which is present in the original def2-TZVP) will decrease the energy of 0.015 eV; further addition of 2f1g functions (as in the def2-QZVP basis set) decreases the energy of only 7 meV.

For Au adding one (reoptimized) f-type function (which is present in the original def2-TZVP) will decrease the energy of 0.049 eV; further addition of 2f1g functions (as in the def2-QZVP basis set) decreases the energy of only 5 meV.

Tab. S11 shows that only for Au one additional f function can be relevant. Thus we tested the lattice constants and cohesive energies for this basis set, for some functionals. Results are reported in Tab. S12 and Tab. S13.

TABLE S11. From left to right: system, added functions with respect the DGBO-MTZ one (i.e. the first line of each block), reoptimized exponents, condition number of the overlap matrix ($\epsilon_{max}/\epsilon_{min}$); conditioned energy ($E_{cond}$) in a.u.; HF total energy (E_{HF}) in a.u.; HF total energy deviation in eV (from the DGBO-MTZ energy).

system	added	expon.	$\epsilon_{max}/\epsilon_{min}$	E_{cond}	E_{HF}	ΔE_{HF} (eV)
Li	-	-	33.401	-0.21236072	-0.21586933	-
Li	2s1p1d	all. reopt	35.165	-0.21234942	-0.21590948	-0.001
Li	1d→2d,1f	0.760 0.190, 0.500	35.876	-0.21233010	-0.21591020	-0.001
Na	-	-	61.123	-0.18392411	-0.18803700	-
Na	1s1p1d	all. reopt.	69.345	-0.18392648	-0.18816558	-0.003
Na	1d→ 3d,1f	3.01 0.502 0.090, 0.315	69.058	-0.18387091	-0.18810586	-0.002
K	-	-	250.445	-28.03433697	-28.03986021	-
K	1f	0.510	253.277	-28.03432846	-28.03986295	-0.000
Al	-	-	188.211	-1.92816934	-1.93340691	-
Al	1f	0.350	245.223	-1.92889771	-1.93439988	-0.027
Al	1f, 1g	0.323, 0.708	249.589	-1.92892898	-1.93444880	-0.028
Cu	-		1658.462	-196.22890928	-196.23632293	-
Cu	1f	1.80	1725.314	-196.22898351	-196.23643667	-0.003
Cu	2f, 1g	4.30 2.00, 0.705	1729.041	-196.22920598	-196.23666130	-0.009
Ag	-	-	1347.969	-146.12599113	-146.13319749	-
Ag	1f	0.982	1348.413	-146.12654355	-146.13375023	-0.015
Ag	3f, 1g	20.0 4.80 0.929, 0.564	1402.872	-146.12676996	-146.13401624	-0.022
Au	-	-	1089.984	-134.80832712	-134.81532104	-
Au	1f	0.801	1381.050	-134.80988425	-134.81711485	-0.049
Au	3f, 1g	3.20 2.12 0.799, 0.911	1611.431	-134.80992106	-134.81730593	-0.054

	HF	revISI	genDPI2-ePC
DGBO-MTZ	4.363	4.040	4.121
DGBO-MTZ+f	4.350	4.029	4.111

TABLE S12. Lattice constants of Au with and without one (optimized) f-function, for different methods.

	HF	revISI	genDPI2-ePC
DGBO-MTZ	0.9965	33.1556	4.3145
DGBO-MTZ+f	1.0221	33.1232	4.2854

TABLE S13. Cohesive energies of Au with and without one (optimized) f-function, for different methods.

S8. SECOND ORDER GRADIENT EXPANSION CORRELATION COEFFICIENT

Let us consider a small perturbation of the spin-unpolarized UEG, such that the density is

$$\begin{aligned} n(\mathbf{r}) &= n_0 + \delta n(\mathbf{r}), \\ \delta n(\mathbf{r}) &\ll |\nabla n| \ll n_0, \\ \int d\mathbf{r}\ \delta n(\mathbf{r}) &= 0. \end{aligned} \tag{S31}$$

Here $n_0 = 3/[4\pi r_s^3]$ is the bulk density, with r_s being the bulk parameter. For such a slowly-varying density, the second-order gradient expansion (GE2) of the correlation energy becomes exact

$$E_c^{GE2}[n] = E_c^{LDA}[n] + \int d\mathbf{r}\ n(\mathbf{r})\beta(r_s)t^2 \tag{S32}$$

$$= E_c^{LDA}[n] + B_c\beta(r_s)I_2[n], \tag{S33}$$

where $t = |\nabla n|/[4(3/\pi)^{1/6}n^{7/6}]$ is the reduced gradient for the correlation and $\beta(r_s)$ is the GE2 correlation coefficient and

$$I_2[n] = \int d\mathbf{r}\ |\nabla n(\mathbf{r})|^2/n(\mathbf{r})^{4/3}, \tag{S34}$$

$$B_c = \pi/[16(3\pi^2)^{1/3}]. \tag{S35}$$

Ma and Brueckner [34] derived β in the high-density limit $\beta(r_s \to 0) = 0.066725$ and this value has been successfully used in the popular PBE functional [16]. Finally, we recall that Hu and Langreth [35] derived the density dependence of β beyond RPA, and their results have been parameterized in Eq. (3) of Ref. 36.

Using Eq. (S31), we can express the strong-interaction functionals as:

$$\begin{aligned} E_x &= A_x I_0[n_0] + B_x I_2[n], \\ W_\infty &= A I_0[n_0] + B_\infty I_2[n], \\ W'_\infty &= C I_3[n_0] + D_\infty I_4[n], \end{aligned} \tag{S36}$$

where

$$\begin{aligned} I_0[n] &= \int d\mathbf{r}\ n^{4/3}(\mathbf{r}), \\ I_3[n] &= \int d\mathbf{r}\ n^{3/2}(\mathbf{r}), \\ I_4[n] &= \int d\mathbf{r}\ |\nabla n(\mathbf{r})|^2/n^{7/6}(\mathbf{r}), \end{aligned} \tag{S37}$$

and

$$\begin{aligned} A_x &= -\frac{3(3\pi^2)^{1/3}}{4\pi}, \\ B_x &= -\frac{A_x\mu_x}{4(3\pi^2)^{2/3}}, \quad \mu_x = 10/81, \\ B_\infty &= -\frac{3}{350}(\frac{3}{4\pi})^{1/3}, \end{aligned} \tag{S38}$$

while for D_∞ we use the revPC value ($D_\infty = -0.028957$) and the ePC value ($D_\infty = 0.0197$), respectively.

Noting that

$$\begin{aligned} I_0[n_0] &= n_0^{4/3}V, \\ I_3[n_0] &= n_0^{3/2}V, \\ I_4[n] &= n_0^{1/6}I_2, \end{aligned} \tag{S39}$$

The correlation coefficient, can also be written as

$$\beta(r_s) = \frac{1}{B_c}\Big(\frac{dE_c}{dI_2}\Big)\Big|_{I_2=0}, \tag{S40}$$

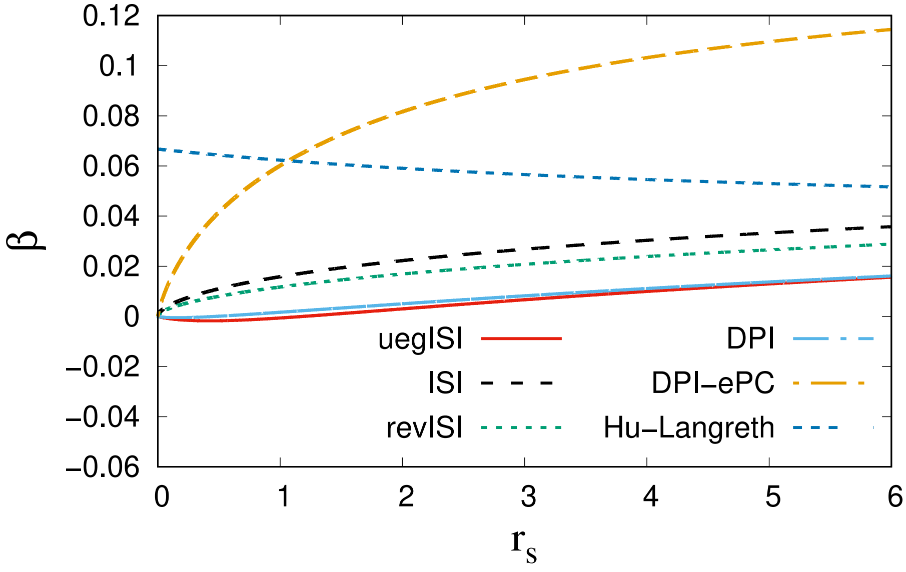

FIG. S2. The GE2 correlation coefficient β (see Eq. (S40)) versus the bulk parameter r_s for the ISI, revISI, uegISI, DPI, and DPI-ePC functionals.

such that it can be easily computed for all the ACII correlation functionals.

In Fig. S2, we show the GE2 coefficient $\beta(r_s)$ of the ISI, revISI, uegISI, DPI and DPI-ePC functionals. For comparison, we also report the accurate interpolation of the Hu and Langreth results [35, 36].

When $r_s \to 0$, all ACII functionals give $\beta \approx 0$, not recovering the Ma and Brueckner [34] coefficient $\beta = 0.066725$ of the high-density limit.

For small $r_s \lesssim 1$ uegISI and DPI are slightly negative, all the other ACII are positive. Comparing the DPI and DPI-ePC curves, we note that β is strongly dependent on the value of D_∞.

In the metallic range $2 \leq r_s \leq 6$, the correlation coefficients of all ACII are smaller than the Hu-Langreth estimate, but genDPI2-ePC which is greater.

At large r_s, the β coefficients of all the ACII correlation functionals behave as

$$\beta(r_s \to \infty) \longrightarrow \frac{B_\infty - B_x}{B_c} \approx 0.12 \,, \tag{S41}$$

being somewhat closer to the non-local coefficient $\beta^{nl} \approx 0.101$ found in Ref. 37 than to the value found from the LDA linear response ($\beta = 0.0375$) used in Ref. 36.

S9. BULK MODULI

TABLE S14. Bulk moduli in GPa for all systems and methods. Last two lines report the mean relative error (MRE) and the mean absolute relative error (MARE) in percent. For K, the ISI equilibrium lattice constant is too small: thus the HF calculations is not converging and the bulk modulus cannot be estimated correctly.

system	HF @HF	PBE @PBE	r^2SCAN @r^2SCAN	HFLDA @HF	HFPBE @HF	HFr^2SCAN @HF	ISI @HF	revISI @HF	uegISI @HF	DPI @HF	DPI-ePC @HF	Ref.[a]
Li	15.7 (9.0[b])	17.3 (14.1[d])	17.7(13.1[e])	17.3	17.5	16.5	19.1	18.4	17.1	17.2	17.5	13.1
Na	7.2 (5[c])	8.3 (7.7[d])	8.5(8.0[e])	8.3	8.6	8.3	9.3	8.8	8.0	8.0	8.6	7.9
K	3.0 (.)	3.8 (3.6[d])	4.0(3.4[e])	3.7	3.9	3.8	-	8.3	4.1	4.2	4.6	3.8
Al	75.3 (80.0[b])	70.0 (77.6[d])	76.3 (93.3[e])	94.0	100.9	97.5	107.8	100.7	80.0	82.9	93.1	77.1
Cu	32.4 (32[c])	130.5 (139.4[d])	153.7 (162.8[f])	46.8	84.3	65.5	164.2	118.5	68.6	71.5	78.1	144.3
Ag	31.7 (27[c])	89.7 (91.0[d])	102.2(104.4[f])	44.2	79.7	65.3	136.1	110.1	66.3	68.3	77.9	105.7
Au	68.7 (.)	133.8 (139.7[d])	147.2 (153.5[f])	85.5	152.3	134.6	209.1	179.5	122.5	127.5	144.0	182.0
MRE(%)	-31.7	-3.3	4.4	-17.7	-1.1	-8.8	107.6[g]	26.7	-11.1	-9.3	-1.1	
MARE(%)	37.3	13.9	11.1	34.4	22.5	25.3	107.6[g]	32.2	23.8	23.8	25.5	

[a] from Ref. 29, [b] from Ref. 30, [c] EXX from PBE orbitals, from Ref. 31, [d] from Ref. 18 [e] from Ref. 22, [f] from Ref. 21, [g] without K.

S10. DGBO-MTZ BASIS SET

Optimized basis sets in CRYSTAL23 format; the stars indicate the functions which have been optimized.

```
-------------------  Li_DGBO ------------------
203 6
INPUT
1. 0 1 1 0 0 0
1.276000 5.786000 0
1.607000 -1.065000 0
0 0 1 1 1.0 *
     4.10E+00 1.0
0 0 1 0 1.0 *
     5.30E-01 1.0
0 0 1 0 1.0 *
     6.40E-02 1.0
0 2 1 0 1.0 *
      1.80E+00 1.0
0 2 1 0 1.0 *
      2.00E-01   1.0
0 3 1 0  1.0 *
       1.60E-01  1.0
99 0
-------------------  Na_DGBO ------------------
211 6
INPUT
1. 0 1 1 1 0 0
1.378000 10.839000 0
0.663900 2.303000 0
0.924900 -1.777000 0
0 0 1 1. 1. *
      3.20E+00 1.0
0 0 1 0. 1. *
      4.60E-01 1.0
0 0 1 0. 1. *
      5.90E-02 1.0
0 2 1 0. 1. *
      7.90E-01 1.0
```

```
0 2 1 0. 1. *
      1.10E-01 1.0
0 3 1 0. 1. *
      8.60E-02 1.0
99 0
-------------------  K_DGBO ------------------
219 10
INPUT
9. 0 1 1 1 1 0
6.843807 92.252639 0
4.217857 23.498035 0
6.497537 -7.315420 0
6.123613 -3.858320 0
0 0  3  2. 1.
      8.22336200000E+00   1.04145000000E-01
      3.79721100000E+00  -4.39158000000E-01
      1.33160700000E+00   5.21910000000E-02
0 0  1  1. 1. *
    6.57E-01     1.00000000000E+00
0 0  1  0. 1. *
   2.61E-01      1.00000000000E+00
0 0   1  0. 1. *
   2.90E-02     1.00000000000E+00
0 2  3  6. 1.
      2.16056700000E+01  -1.17370000000E-02
      1.10021200000E+00  -4.07675000000E-01
      5.04345000000E-01  -3.33292000000E-01
0 2  1  0. 1. *
    2.91E-01    1.00000000000E+00
0 2  1  0. 1. *
    9.51E-02    1.00000000000E+00
0 3  1  0. 1. *
    1.80E+00        1.00000000000E+00
0 3  1  0. 1. *
   3.46E-01     1.00000000000E+00
0 3  1  0. 1. *
   5.00E-02      1.00000000000E+00
99 0
-------------------  Al_DGBO ------------------
213 10
INPUT
3 0 1 1 1 0 0
2.198225000 20.409813000 0
1.601395000 8.980495000 0
1.499026000 -1.970411000 0
0 0  1  2. 1. *
     4.50E+00 1.0
0 0  1  0  1. *
     1.10E+00 1.0
0 0  1  0. 1. *
     4.21E-01 1.0
0 0  1  0. 1. *
     1.60E-01 1.0
0 2  1  1. 1. *
     5.41E+00 1.0
0 2  1  0  1. *
     2.31E+00 1.0
0 2  1  0  1. *
```

```
     1.10E+00 1.0
0 2  1  0  1. *
     1.88E-01 1.0
0 3  1  0. 1. *
    7.04E-01 1.0
0 3  1  0. 1. *
    1.70E-01 1.0
99 0
-------------------  Cu_DGBO ------------------
229 13
INPUT
19. 0 2 2 2 0 0
30.220000 355.770158 0
13.190000 70.865357 0
33.130000 233.891976 0
13.220000 53.947299 0
38.420000 -31.272165 0
13.260000 -2.741104 0
0 0  3  2. 1.
      2.76743324800E+01   2.31132000000E-01
      1.36163446942E+01  -6.56811000000E-01
      9.09453748276E+00  -5.45875000000E-01
0 0  1  1. 1. *
     3.29E+00    1.00000000000E+00
0 0  1  0. 1. *
     1.99E+00    1.00000000000E+00
0 0  1  0. 1. *
     8.70E-01    1.00000000000E+00
0 0   1  0. 1. *
     1.79E-01    1.00000000000E+00
0 2  2  6. 1.
      9.35042020229E+01   2.28290000000E-02
      1.62063239460E+01  -1.00951300000E+00
0 2  2  0. 1.
      5.80371320215E+00   2.46450000000E-01
      2.49129816765E+00   7.92024000000E-01
0 2  1  0. 1. *
      9.30E-01   1.00000000000E+00
0 2  1  0. 1. *
      5.70E-01   1.00000000000E+00
0 2  1  0. 1.  *
      1.92E-01   1.00000000000E+00
0 3  5 10. 1.
      8.16999747268E+01   9.05000000000E-03
      2.65000187233E+01   6.07000000000E-02
      1.00052862739E+01   1.82000000000E-01
      4.11190447429E+00   3.16000000000E-01
      1.67612230258E+00   3.65000000000E-01
0 3  1  0. 1. *
      6.48E-01   1.00000000000E+00
0 3  1  0. 1. *
      2.20E-01  1.00000000000E+00
99 0
-------------------  Ag_DGBO ------------------
247 12
INPUT
19. 0 2 2 2 2 0
13.130000 255.139365 0
```

```
6.510000 36.866122 0
11.74000 182.181869 0
6.200000 30.357751 0
10.210000 73.719261 0
4.380000 12.502117 0
14.220000 -33.689920 0
7.110000 -5.531120 0
0 0  2  2. 1.
      1.90000000000E+01  -1.66001041410E-01
      1.54281999330E+01   3.56650959180E-01
0 0  1  1. 1. *
      5.80E+00  1.00000000000E+00
0 0  1  0. 1. *
      2.40E+00   1.00000000000E+00
0 0  1  0. 1. *
      1.10E+00   1.00000000000E+00
0 0  1  0. 1. *
      3.29E-01   1.00000000000E+00
0 0   1  0. 1. *
      1.59E-01   1.00000000000E+00
0 2  4  6. 1.
      1.31881801800E+01   6.69287371470E-02
      7.79527891380E+00  -2.47352354090E-01
      2.03515719120E+00   4.91542802160E-01
      0.98093914842E+00   0.49741609006
0 2  1  0. 1. *
      4.49E-01 1.00000000000E+00
0 2  1  0. 1. *
      1.38E-01 1.00000000000E+00
0 3  4 10. 1.
      2.57843973510E+01   3.56450630800E-03
      1.13966367550E+01  -1.29842627840E-02
      2.73455813610E+00   2.41088265480E-01
      1.18735836050E+00   4.24123307440E-01
0 3  1  0. 1. *
      4.88E-01 1.00000000000E+00
0 3  1  0. 1. *
      1.76E-01 1.00000000000E+00
99 0
-------------------  Au_DGBO ------------------
279 12
INPUT
19. 0 2 2 2 2 0
13.205100 426.846679 0
6.602550 37.007083 0
10.452020 261.199580 0
5.226010 26.962496 0
7.851100 124.790666 0
3.925550 16.300726 0
4.789820 30.490089 0
2.394910 5.171074 0
0 0  3  2. 1.
      3.00000000000E+01   2.07492311080E-01
      2.70000000000E+01  -3.32678933940E-01
      1.47468243310E+01   3.83028179580E-01
0 0  1  1. 1. *
       5.60E+00  1.00000000000E+00
0 0  1  0. 1. *
```

```
      1.40E+00  1.00000000000E+00
0 0  1  0. 1. *
      6.55E-01  1.00000000000E+00
0 0   1  0. 1. *
      1.49E-01   1.00000000000E+00
0 2  4  6. 1.
      1.55000000000E+01   1.50017118800E-01
      1.40000000000E+01  -2.36098131830E-01
      6.42273682050E+00   3.14588969480E-01
      1.65956016810E+00  -5.72796704460E-01
0 2  1  0. 1. *
      8.91E-01  1.00000000000E+00
0 2  1  0. 1. *
      5.82E-01  1.00000000000E+00
0 2  1  0. 1. *
     1.40E-01   1.00000000000E+00
0 3  4 10. 1.
      9.55240986560E+00   4.01455595020E-02
      7.26988869370E+00  -9.36909066060E-02
      1.77464967890E+00   3.17462823170E-01
      7.99605410550E-01   4.67951924830E-01
0 3  1  0. 1.  *
     4.02E-01  1.00000000000E+00
0 3  1  0. 1.  *
     2.08E-01   1.00000000000E+00
99 0
```

S11. BAND-STRUCTURE AND DOS

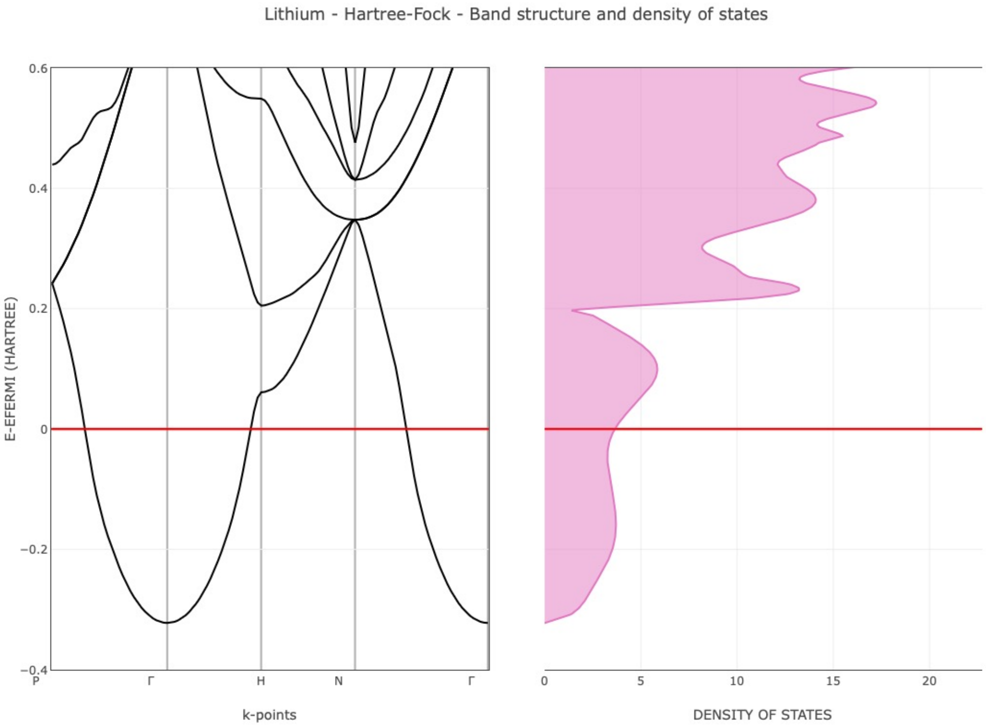

FIG. S3.

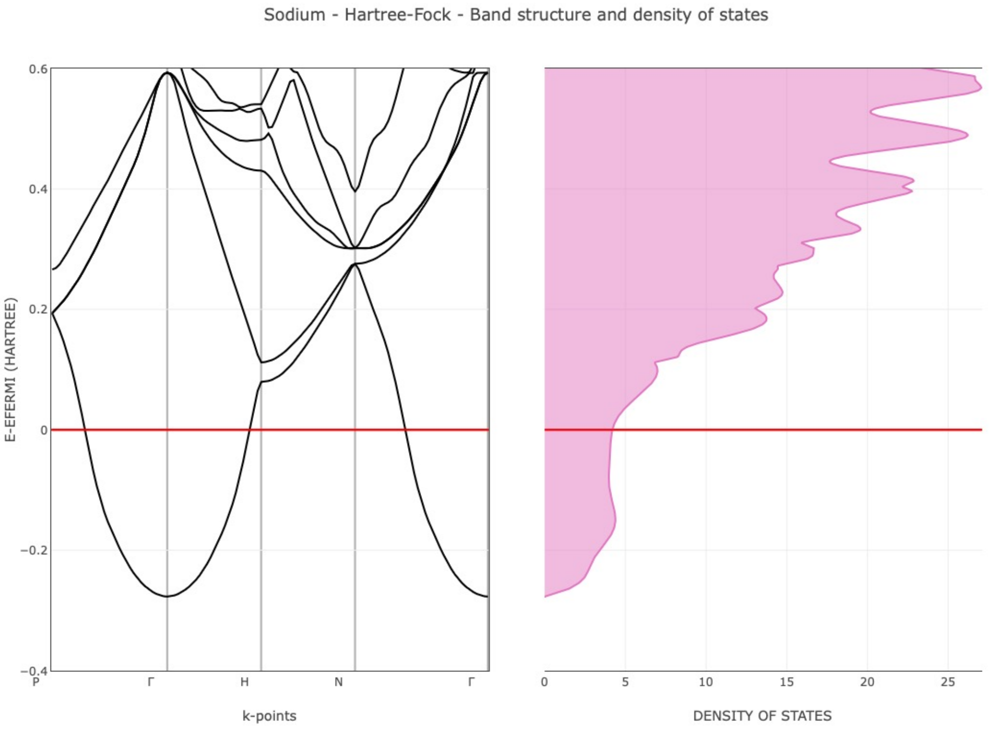
Sodium - Hartree-Fock - Band structure and density of states
E-EFERMI (HARTREE)
0.6
0.4
0.2
0
−0.2
−0.4
P
Γ
H
N
Γ
k-points
0
5
10
15
20
25
DENSITY OF STATES

FIG. S4.

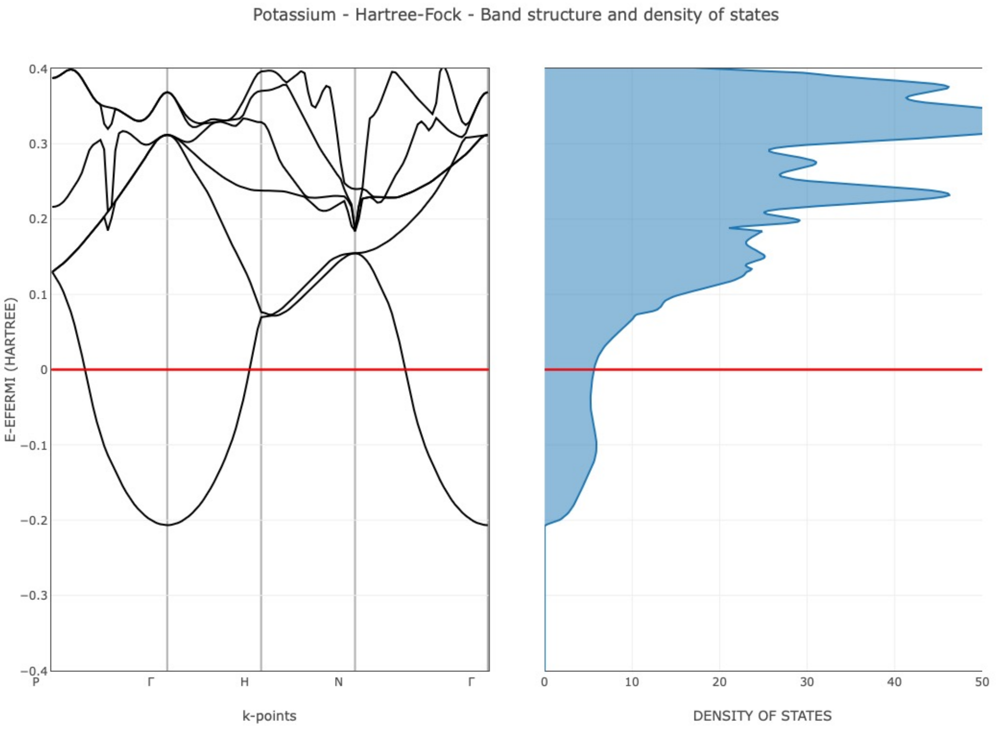
Potassium - Hartree-Fock - Band structure and density of states
E-EFERMI (HARTREE)
0.4
0.3
0.2
0.1
0
−0.1
−0.2
−0.3
−0.4
P
Γ
H
N
Γ
k-points
0
10
20
30
40
50
DENSITY OF STATES

FIG. S5.

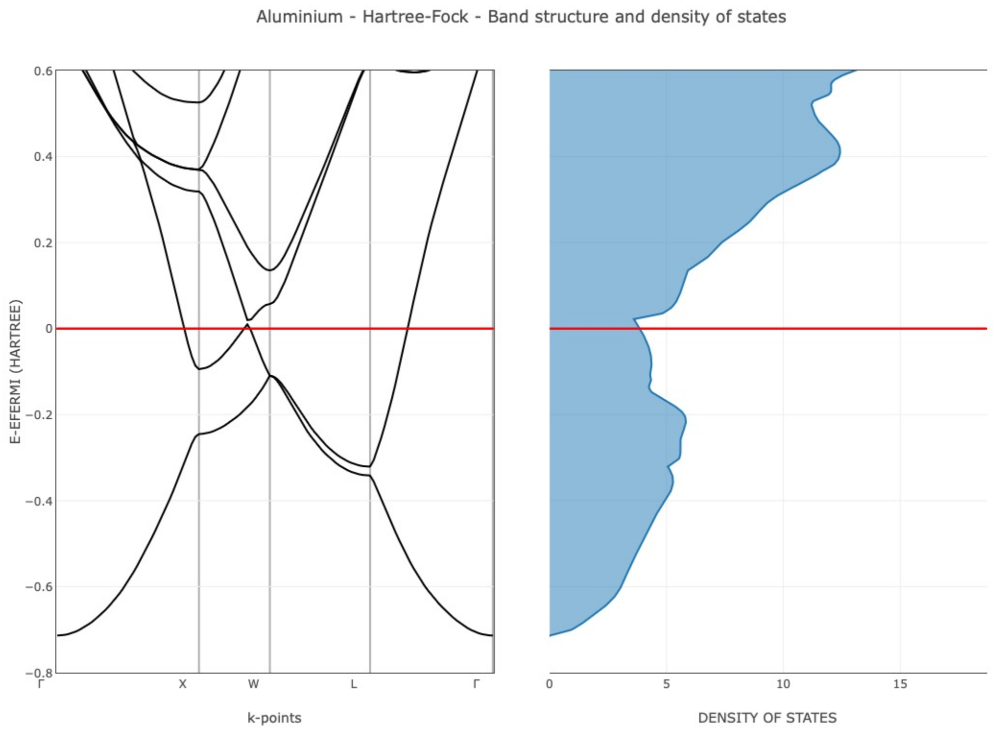

Aluminium - Hartree-Fock - Band structure and density of states
E-EFERMI (HARTREE)
k-points
DENSITY OF STATES
Γ
X
W
L
Γ

FIG. S6.

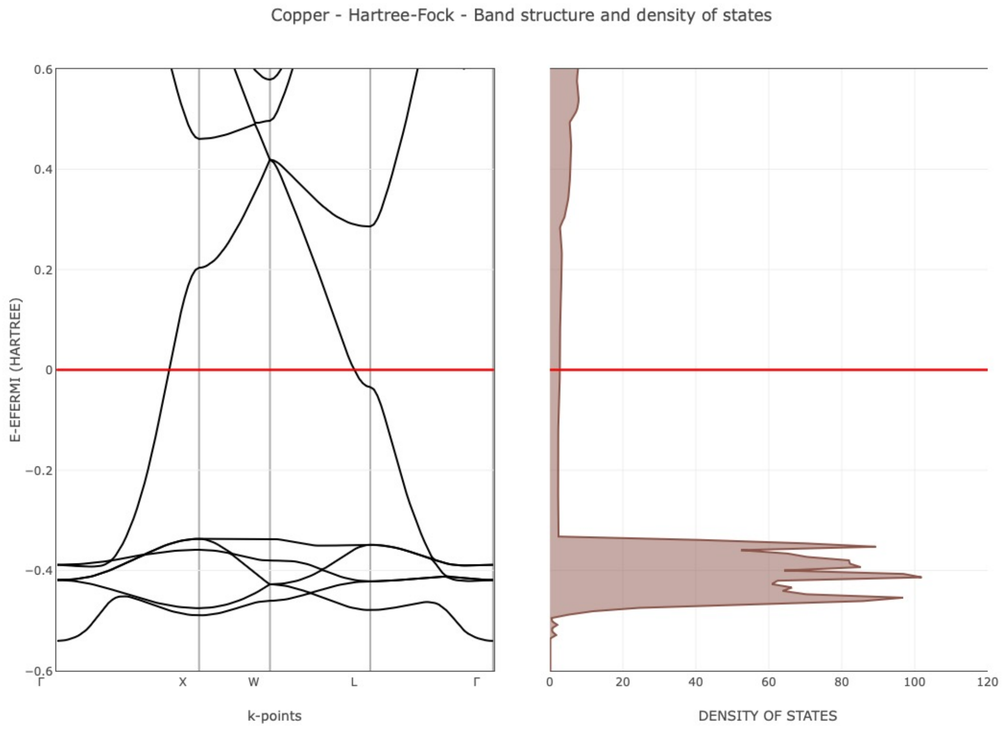

Copper - Hartree-Fock - Band structure and density of states
E-EFERMI (HARTREE)
k-points
DENSITY OF STATES
Γ
X
W
L
Γ

FIG. S7.

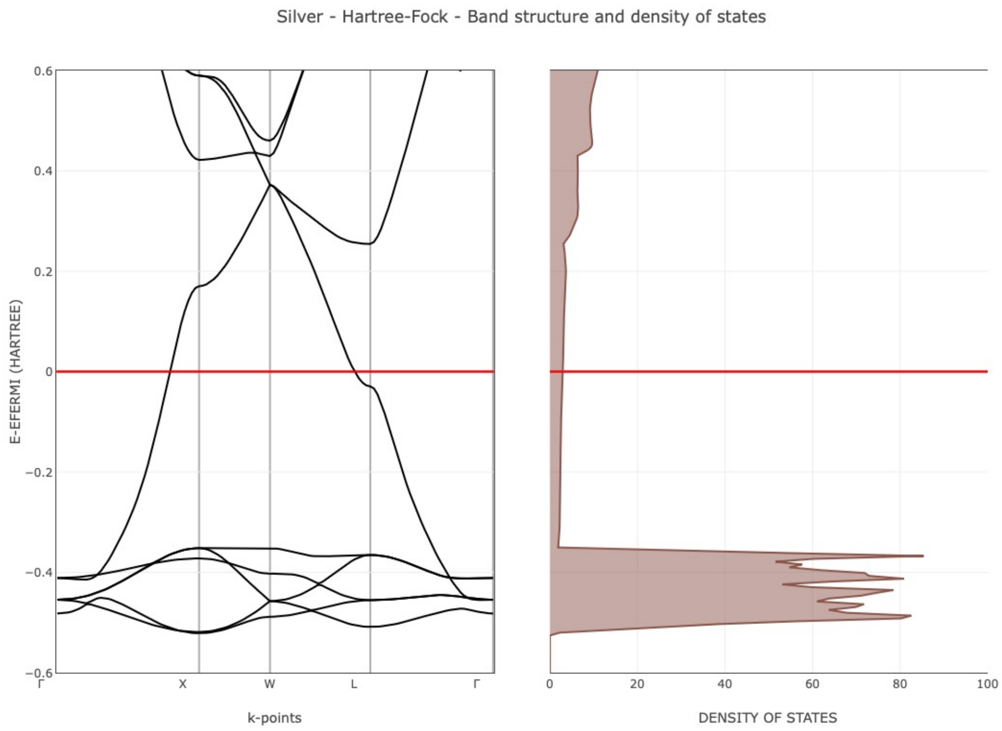

Silver - Hartree-Fock - Band structure and density of states
E-EFERMI (HARTREE)
0.6
0.4
0.2
0
−0.2
−0.4
−0.6
Γ
X
W
L
Γ
k-points
0
20
40
60
80
100
DENSITY OF STATES

FIG. S8.

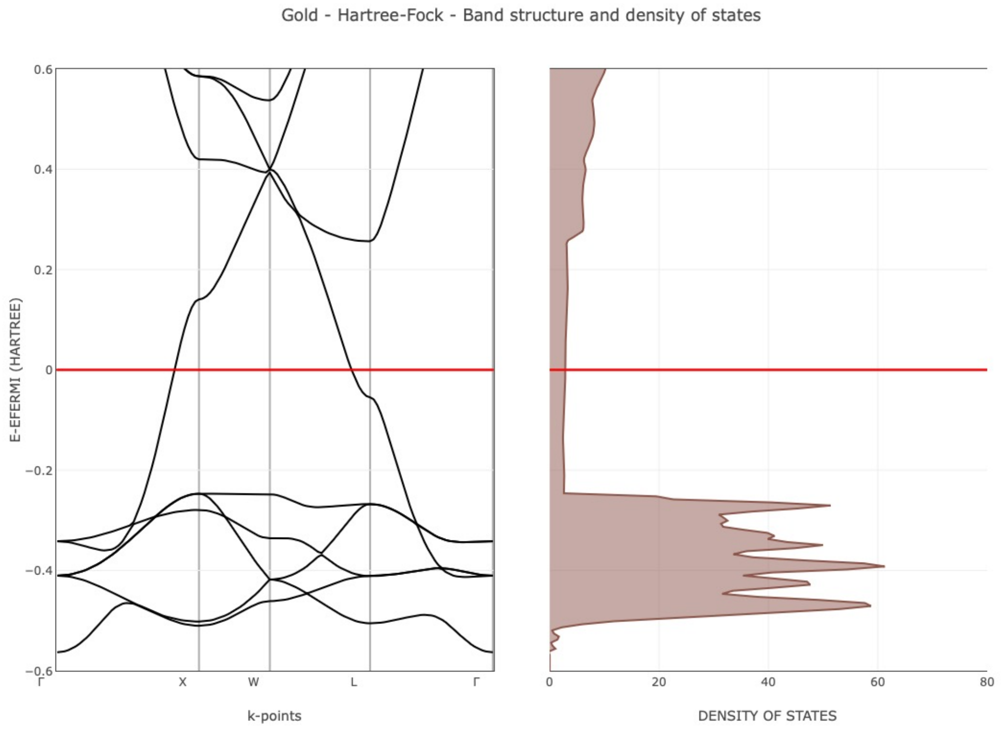

Gold - Hartree-Fock - Band structure and density of states
E-EFERMI (HARTREE)
0.6
0.4
0.2
0
−0.2
−0.4
−0.6
Γ
X
W
L
Γ
k-points
0
20
40
60
80
DENSITY OF STATES

FIG. S9.

[1] M. Seidl, J. P. Perdew, and S. Kurth, Simulation of all-order density-functional perturbation theory, using the second order and the strong-correlation limit, Phys. Rev. Lett. **84**, 5070 (2000).
[2] P. Gori-Giorgi, G. Vignale, and M. Seidl, Electronic zero-point oscillations in the strong-interaction limit of density functional theory, J. Chem. Theory Comput. **5**, 743 (2009).
[3] L. A. Constantin, S. Jana, S. Śmiga, and F. Della Sala, Adiabatic connection interaction strength interpolation method made accurate for the uniform electron gas, J. Chem. Phys. **159**, 244111 (2023).
[4] J. Sun, J. P. Perdew, and M. Seidl, Correlation energy of the uniform electron gas from an interpolation between high- and low-density limits, Phys. Rev. B **81**, 085123 (2010).
[5] M. Seidl, J. P. Perdew, and S. Kurth, Density functionals for the strong-interaction limit, Phys. Rev. A **62**, 012502 (2000).
[6] A. Erba, J. K. Desmarais, S. Casassa, B. Civalleri, L. Donà, I. J. Bush, B. Searle, L. Maschio, L. Edith-Daga, A. Cossard, C. Ribaldone, E. Ascrizzi, N. L. Marana, J.-P. Flament, and B. Kirtman, Crystal23: A program for computational solid state physics and chemistry, J. Chem. Theory Comput. **19**, 6891 (2023).
[7] R. Dovesi, V. Saunders, C. Roetti, R. Orlando, C. Zicovich-Wilson, F. Pascale, B. Civalleri, K. Doll, N. Harrison, I. Bush, P. D'Arco, M. Llunell, M. Causà, Y. Noël, L. Maschio, A. Erba, M. Rerat, S. Casassa, B. Searle, and J. K. Desmarais, Crystal23 user's manual, (University of Torino, Torino (2023).
[8] S. Śmiga, F. Della Sala, P. Gori-Giorgi, and E. Fabiano, Self-consistent implementation of kohn-sham adiabatic connection models with improved treatment of the strong-interaction limit, J. Chem. Theory Comput. **18**, 5936 (2022).
[9] L. A. Constantin, F. Naeem, E. Fabiano, F. Sarcinella, and F. Della Sala, Restoring the point-and-charge gradient expansion for strong interaction density functionals, Phys. Rev. B **113**, 085121 (2026).
[10] E. Fabiano, `https://github.com/e-fabiano/acmxc` (2024), accessed: 2024-11-25.
[11] F. Sarcinella, F. Della Sala, and E. Fabiano, `https://github.com/ful-sar/cryslatt` (2024), accessed: 2024-11-25.
[12] M. Dolg, U. Wedig, H. Stoll, and H. Preuss, Energy-adjusted ab initio pseudopotentials for the first row transition elements, The Journal of Chemical Physics **86**, 866 (1987).
[13] D. Andrae, U. Häußermann, M. Dolg, H. Stoll, and H. Preuß, Energy-adjustedab initio pseudopotentials for the second and third row transition elements, Theoretica chimica acta **77**, 123 (1990).
[14] M. Kaupp, P. v. R. Schleyer, H. Stoll, and H. Preuss, Pseudopotential approaches to ca, sr, and ba hydrides. why are some alkaline earth mx2 compounds bent?, The Journal of Chemical Physics **94**, 1360 (1991).
[15] T. Leininger, A. Nicklass, W. Küchle, H. Stoll, M. Dolg, and A. Bergner, The accuracy of the pseudopotential approximation: non-frozen-core effects for spectroscopic constants of alkali fluorides xf (x = k, rb, cs), Chemical Physics Letters **255**, 274 (1996).
[16] J. P. Perdew, K. Burke, and M. Ernzerhof, Generalized gradient approximation made simple, Phys. Rev. Lett. **77**, 3865 (1996).
[17] J. W. Furness, A. D. Kaplan, J. Ning, J. P. Perdew, and J. Sun, Accurate and numerically efficient r2scan meta-generalized gradient approximation, Jou. Phys. Chem. Lett. **11**, 8208 (2020).
[18] G.-X. Zhang, A. M. Reilly, A. Tkatchenko, and M. Scheffler, Performance of various density-functional approximations for cohesive properties of 64 bulk solids, New. Jou. Phys. **20**, 063020 (2018).
[19] P. Kovács, F. Tran, P. Blaha, and G. K. H. Madsen, Comparative study of the pbe and scan functionals: The particular case of alkali metals, J. Chem. Phys. **150**, 164119 (2019).
[20] P. Haas, F. Tran, P. Blaha, L. S. Pedroza, A. J. R. da Silva, M. M. Odashima, and K. Capelle, Systematic investigation of a family of gradient-dependent functionals for solids, Phys. Rev. B **81**, 125136 (2010).
[21] H. Liu, X. Bai, J. Ning, Y. Hou, Z. Song, A. Ramasamy, R. Zhang, Y. Li, J. Sun, and B. Xiao, Assessing r2scan meta-gga functional for structural parameters, cohesive energy, mechanical modulus, and thermophysical properties of 3d, 4d, and 5d transition metals, J. Chem. Phys. **160**, 024102 (2024).
[22] A. D. Kaplan and J. P. Perdew, Laplacian-level meta-generalized gradient approximation for solid and liquid metals, Phys. Rev. Mater. **6**, 083803 (2022).
[23] F. Weigend and R. Ahlrichs, Balanced basis sets of split valence, triple zeta valence and quadruple zeta valence quality for h to rn: Design and assessment of accuracy, Phys. Chem. Chem. Phys. **7**, 3297 (2005).
[24] B. P. Pritchard, D. Altarawy, B. Didier, T. D. Gibson, and T. L. Windus, New basis set exchange: An open, up-to-date resource for the molecular sciences community, Journal of Chemical Information and Modeling **59**, 4814 (2019).
[25] G. Wang, A. Annaberdiyev, C. A. Melton, M. C. Bennett, L. Shulenburger, and L. Mitas, A new generation of effective core potentials from correlated calculations: 4s and 4p main group elements and first row additions, J. Chem. Phys. **151**, 144110 (2019).
[26] G. Wang, B. Kincaid, H. Zhou, A. Annaberdiyev, M. C. Bennett, J. T. Krogel, and L. Mitas, A new generation of effective core potentials from correlated and spin–orbit calculations: Selected heavy elements, J. Chem. Phys. **157**, 054101 (2022).
[27] E. C. Barnes and G. A. Petersson, Mp2/cbs atomic and molecular benchmarks for h through ar, J. Chem. Phys. **132**, 114111 (2010).
[28] B. Paulus and K. Rosciszewski, Hartree–fock ground-state properties for the group 1 alkali metals and the group 11 noble metals, J. Phys.: Cond. Matt. **19**, 346217 (2007).
[29] K. Lejaeghere, V. V. Speybroeck, G. V. Oost, and S. Cottenier, Error estimates for solid-state density-functional theory predictions: An overview by means of the ground-state elemental crystals, Critical Reviews in Solid State and Materials

Sciences **39**, 1 (2014).
[30] V. A. Neufeld, H.-Z. Ye, and T. C. Berkelbach, Ground-state properties of metallic solids from ab initio coupled-cluster theory, J. Phys. Chem. Lett. **13**, 7497–7503 (2022).
[31] J. Harl, L. Schimka, and G. Kresse, Assessing the quality of the random phase approximation for lattice constants and atomization energies of solids, Phys. Rev. B **81**, 115126 (2010).
[32] H.-Z. Ye and T. C. Berkelbach, Correlation-consistent gaussian basis sets for solids made simple, J. Chem. Theory Comput. **18**, 1595 (2022).
[33] C. Pisani, R. Dovesi, and C. Roetti, Hartree-Fock ab initio treatment of crystalline systems (Springer Science & Business Media, 2012).
[34] S.-k. MA and K. A. BRUECKNER, Correlation energy of an electron gas with a slowly varying high density, Phys. Rev. **165**, 18 (1968).
[35] C. D. Hu and D. C. Langreth, Beyond the random-phase approximation in nonlocal-density-functional theory, Phys. Rev. B **33**, 943 (1986).
[36] J. P. Perdew, A. Ruzsinszky, G. I. Csonka, L. A. Constantin, and J. Sun, Workhorse semilocal density functional for condensed matter physics and quantum chemistry, Phys. Rev. Lett. **103**, 026403 (2009).
[37] O. A. Vydrov and T. Van Voorhis, Nonlocal van der waals density functional made simple, Phys. Rev. Lett. **103**, 063004 (2009).
